# Determination of the transcription unit landscape and associated regulatory elements in *Methylosinus sporium* 5

**DOI:** 10.1128/spectrum.01281-25

**Published:** 2025-08-06

**Authors:** Jiyun Bae, Dong-Uk Song, Hyewon Lee, Seung-Goo Lee, Byung-Kwan Cho

**Affiliations:** 1 Department of Biological Sciences, Korea Advanced Institute of Science and Technology 34968, Daejeon, Republic of Korea; 2 Graduate School of Engineering Biology, Korea Advanced Institute of Science and Technology 34968, Daejeon, Republic of Korea; 3 Synthetic Biology Research Center and the K-Biofoundry, Korea Research Institute of Bioscience and Biotechnology (KRIBB), Daejeon, Republic of Korea; 4 Department of Biosystems and Bioengineering, KRIBB School of Biotechnology, University of Science & Technology, Daejeon, Republic of Korea; 5 KI for the BioCentury, Korea Advanced Institute of Science and Technology 34968, Daejeon, Republic of Korea; Forschungszentrum Jülich GmbH, Juelich, Germany

**Keywords:** methanotroph, methane, multi-omics, regulatory elements, transcriptome architecture

## Abstract

Methanotrophic bacteria show significant promise for methane bioconversion. Despite their ecological and biotechnological importance, the understanding of their transcriptional regulation and genetic regulatory elements remains limited. Here, we applied high-throughput sequencing to elucidate the transcriptional regulatory landscape of *Methylosinus sporium* 5, a type II methanotroph. With its genome sequence completion, we identified 1,983 transcription start sites (TSSs) and 1,483 transcript 3′-ends (TEPs), which collectively defined 1,431 transcription units (TUs). This comprehensive analysis revealed diverse regulatory elements, including promoters, untranslated regions (UTRs), terminators, and regulatory RNAs in *M. sporium* 5. A consensus promoter motif comprizing conserved −10 (TATAHT) and −35 (TYGMSV) elements, recognized by the housekeeping sigma factor RpoD, was predominant, particularly upstream of genes involved in methane and central carbon metabolism. We also uncovered diverse *cis*-regulatory motifs associated with nitrogen metabolism and cell division, including binding sites for RpoN (σ^54^) and the transcription regulator CtrA. TEP analysis identified three classes of transcript ends with distinct sequence features and termination strengths. I-shaped intrinsic terminators were most prevalent, while L-shaped terminators were enriched in highly expressed genes, ensuring efficient transcription termination. Integration of TSSs and TEPs revealed functionally related genes within polycistronic TUs and identified previously unannotated small RNAs, including EcpR1 and αr45. This study provides the first genome-scale map of transcriptional regulation in a methanotroph, offering foundational insights into regulatory architecture and enabling future strain engineering for enhanced methane bioconversion.

**IMPORTANCE:**

Methanotrophic bacteria offer a sustainable solution for converting methane into valuable products. However, the molecular mechanisms governing gene expression regulation in methanotrophs remain poorly understood. In this study, we applied high-throughput sequencing approaches to elucidate gene organization and transcriptional regulation in *Methylosinus sporium* 5 during growth on methane. By identifying key regulatory features including promoter sequences, diverse *cis*-regulatory elements, and transcript boundaries, we revealed the coordinated gene expression mechanisms in this organism. This represents the first genome-wide transcriptome architecture in a methanotrophic bacterium. The regulatory elements provide a valuable resource for future genetic engineering of *M. sporium* 5 and related methanotrophs. Our findings significantly advance the understanding of gene regulation in methanotrophs and support their development as microbial platforms for methane-based biomanufacturing.

## INTRODUCTION

Methane is the second most abundant anthropogenic greenhouse gas, with a significantly higher global warming potential than carbon dioxide (CO_2_) over the past century (1). While CO_2_ can be naturally captured by plants, algae, and several bacteria (2), methane sequestration is restricted to specialized microorganisms known as methanotrophs (3, 4). As the primary biological sink for atmospheric methane, methanotrophs play a vital role in the global carbon cycle by oxidizing methane through methane monooxygenase (MMO) enzymes (5). Thus, methanotrophs have been harnessed as biocatalysts to produce value-added biochemicals, including lactic acid, 3-hydroxypropionic acid, isoprene, and cadaverine (6–12), through metabolic engineering.

To improve methane bioconversion efficiency, considerable efforts have focused on optimizing gene expression in methanotrophs (13, 14). Strategies such as constructing promoter libraries with a broad range of strengths, ribosome binding site (RBS) libraries, and combinatorial designs of both have enabled precise gene regulation and improved target biochemical production (6, 10, 11, 14). However, despite growing interest in engineering genetic regulatory elements, our understanding of transcriptional regulation in methanotrophs remains limited. Particularly, a comprehensive, genome-wide characterization of native regulatory elements, including promoters, untranslated regions (UTRs), terminators, and regulatory RNAs, has not yet been achieved.

Differential RNA sequencing (dRNA-seq) and Term-seq enable the identification of transcription start sites (TSSs) and transcript 3′-end positions (TEPs), respectively (15, 16), thereby comprehensively elucidating regulatory elements and transcript boundaries (17–19). Although transcriptomic and proteomic studies have revealed dynamic gene expression responses to methane and methanol (20–22), nitrogen source (23), and salt stress (24), genome-wide, single-nucleotide resolution approaches to define TSSs and TEPs have not yet been applied to methanotrophs. Here, we present the first comprehensive analysis of the transcriptome architecture of *Methylosinus sporium* 5 (DSM 17706), a type II methanotroph widely studied for its soluble MMO (sMMO) (25, 26). We first obtained a complete genome sequence to establish the genetic background of *M. sporium*. We then constructed its genome-wide transcription units (TUs) map based on TSS and TEP information obtained from dRNA-Seq and Term-Seq. Our analysis revealed that the housekeeping sigma (σ) factor RpoD predominantly controls gene expression, including genes associated with methane metabolism. In addition, we uncovered diverse *cis*-regulatory elements potentially involved in regulating cellular processes such as cell division, cellular architecture, nitrogen metabolism, and stress responses. We also discovered previously unannotated small RNAs (sRNAs) and distinct terminator sequence features, suggesting complex post-transcriptional regulation in *M. sporium*. Collectively, these findings advance our understanding of transcriptional regulation in methanotrophs and provide a foundational resource for future genetic and metabolic engineering.

## RESULTS

### Complete genome sequence and comparative genomic analysis of *M. sporium* 5

**Fig 1:**
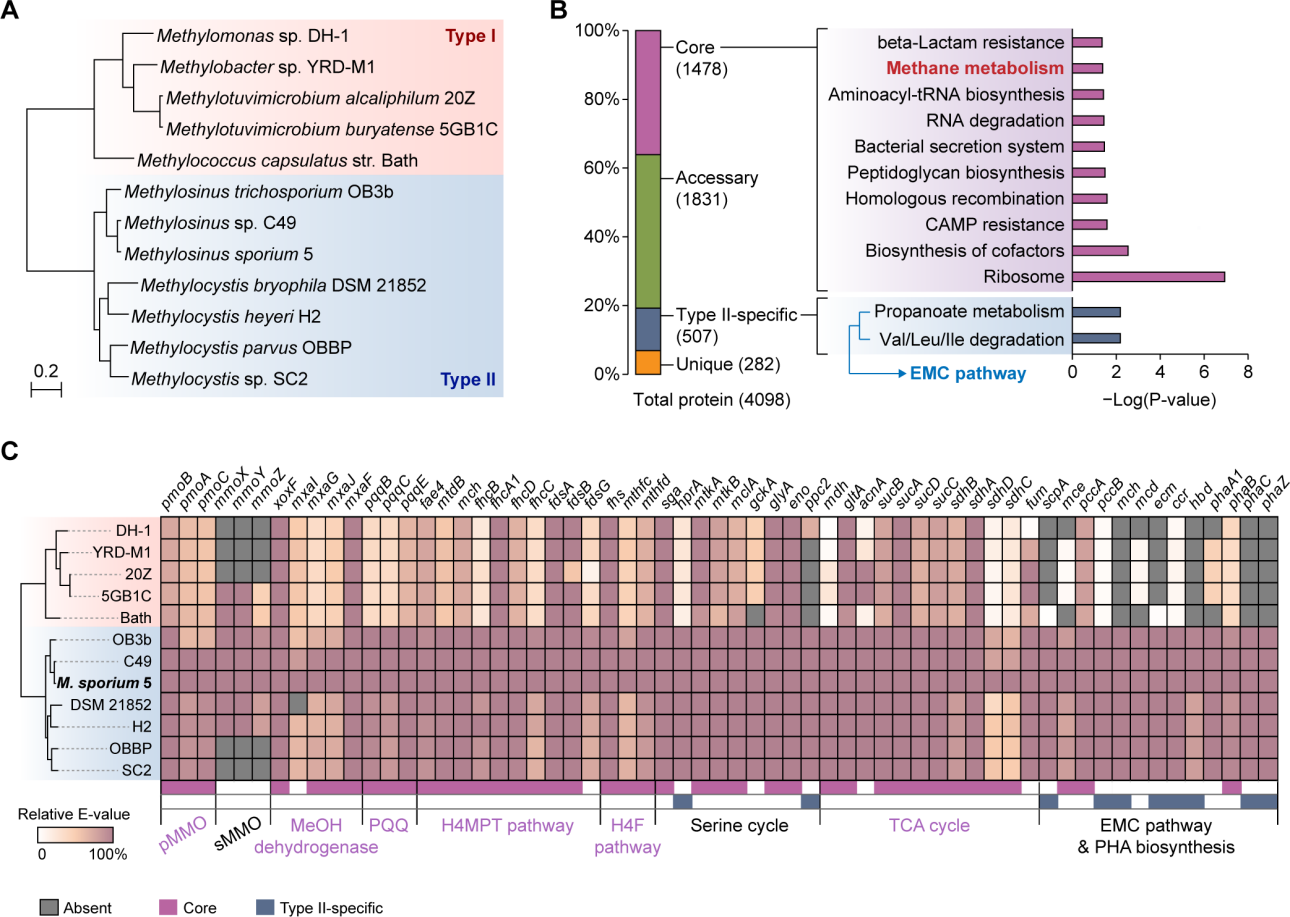
Comparative genomic analysis of *Methylosinus sporium* 5. (**A**) Phylogenetic analysis of *M. sporium* 5 and 11 other methanotrophs with complete genomes. The phylogenetic tree was constructed based on single-copy orthologous proteins identified using Orthofinder (27). Clades representing type I and type II methanotrophs are highlighted in red and blue, respectively. (**B**) Distribution of *M. sporium* 5 proteins into core, type II-specific, accessory, and unique ortholog groups. KEGG pathway enrichment results for each group are shown in the adjacent bar graph. Statistical significance was assessed using the Benjamini–Hochberg correction, with *P* < 0.05 considered significant. Significance values are presented as −log_10_ (*P* value). (**C**) Comparative analysis of proteins involved in methane metabolism across 12 methanotrophs. BLASTP searches were performed using *M. sporium* 5 protein sequences as the reference. The color scale represents relative *E*-values, calculated by dividing the –log(*E*-value) of each homolog by that of the *M. sporium* self-hit for the same protein and expressing the result as a percentage. Corresponding locus tags for the listed gene names are provided in Data S1. CAMP, cationic antimicrobial peptide; EMC, ethylmalonyl-CoA; H4F, tetrahydrofolate; H4MPT, tetrahydromethanopterin; PHA, polyhydroxyalkanoate; pMMO, particulate MMO; PQQ, pyrroloquinoline quinone; sMMO, soluble MMO.

The *M. sporium* 5 genome consists of a circular chromosome (4.1 Mbp) and two plasmids (270 and 116 kbp), totaling 4.5 Mbp with 65% GC content (Table S1). The chromosome encodes 3,966 genes, including 3,906 coding sequences, 6 rRNAs (5S, 16S, 23S), 50 tRNAs, 3 ncRNAs, and 1 tmRNAs. Both plasmids, encoding 173 and 97 coding genes, respectively, harbor the *repABC* operon (MSP_004072-004074 on plasmid 1 and MSP_004218-004220 on plasmid 2), which mediates plasmid replication and segregation in α-proteobacteria, confirming these contigs as bona fide plasmids. The presence of multiple plasmids and high GC content is consistent with genomic features observed in other methanotrophs (Table S2). Genome-based phylogenetic analysis of representative methanotrophs formed two major clades corresponding to type I (γ-proteobacteria) and type II (α-proteobacteria) methanotrophs, placing *M. sporium* 5 within the type II clade, closely related to *Methylosinus* sp. C49 and *Methylosinus trichosporium* OB3b (Fig. 1A).

Comparative analysis of 12 methanotroph genomes identified 5,707 ortholog groups, which were systematically categorized into core (935 groups), type I-specific (313 groups), type II-specific (460 groups), species-specific orthologs, and singletons (Table S3). Among the 4,098 proteins annotated in *M. sporium* genome, 1,478 were assigned to the core ortholog group conserved across methanotroph species (Fig. 1B). Functional enrichment analysis showed that core orthologs included genes involved in methane metabolism and essential cellular processes such as translation and cofactor/peptidoglycan biosynthesis. Particularly, genes responsible for methane conversion to oxidized C1 compounds (formaldehyde, formate, and CO_2_) were highly conserved across methanotrophs. These included genes encoding copper-containing particulate MMO (pMMO), methanol dehydrogenase (*mxa* operon and *xoxF*), pyrroloquinoline quinone (PQQ) biosynthesis, and pathways for formaldehyde and formate oxidation via tetrahydromethanopterin (H4MPT) and tetrahydrofolate (H4F), respectively (Fig. 1C). Interestingly, the iron-containing sMMO was present only in a subset of methanotrophs, including *M. sporium*, which possesses both *pmoABC* (pMMO) and *mmoXYZ* (sMMO) operons, indicating its potential to switch between MMOs depending on copper availability (28).

Assimilation of oxidized C1 compounds into central carbon metabolism differs between type I and type II methanotrophs. Type I utilizes the ribulose monophosphate (RuMP) cycle, whereas type II utilizes the serine cycle (3). As a type II methanotroph, *M. sporium* contained a complete set of genes required for the serine cycle but lacked 3-hexulose-6-phosphate synthase, a key first-step enzyme for the RuMP cycle, confirming its reliance on the serine cycle for formaldehyde assimilation (Fig. 1C). A distinguishing feature of type II methanotrophs was the exclusive presence of the ethylmalonyl-CoA (EMC) pathway, essential for efficient C1 assimilation in type II methanotrophs, which replenishes intermediates of the TCA and serine cycles (succinyl-CoA and glyoxylate, respectively) (29). In *M. sporium*, 507 proteins were classified into type II-specific ortholog groups, including genes involved in propanoate metabolism and valine/leucine/isoleucine degradation, several of which overlap with the EMC pathway (Fig. 1B). Furthermore, type II methanotrophs, including *M. sporium*, possess genes for polyhydroxyalkanoate (PHA) biosynthesis (*phaABC*), which is connected to the EMC pathway (Fig. 1C). The presence of *phaZ*, encoding PHA depolymerase, suggests the capacity for both synthesis and depolymerization of PHA, highlighting the potential of *M. sporium* for carbon and energy storage regulation.

### Transcriptional responses to methane and ammonium supplementation

**Fig 2:**
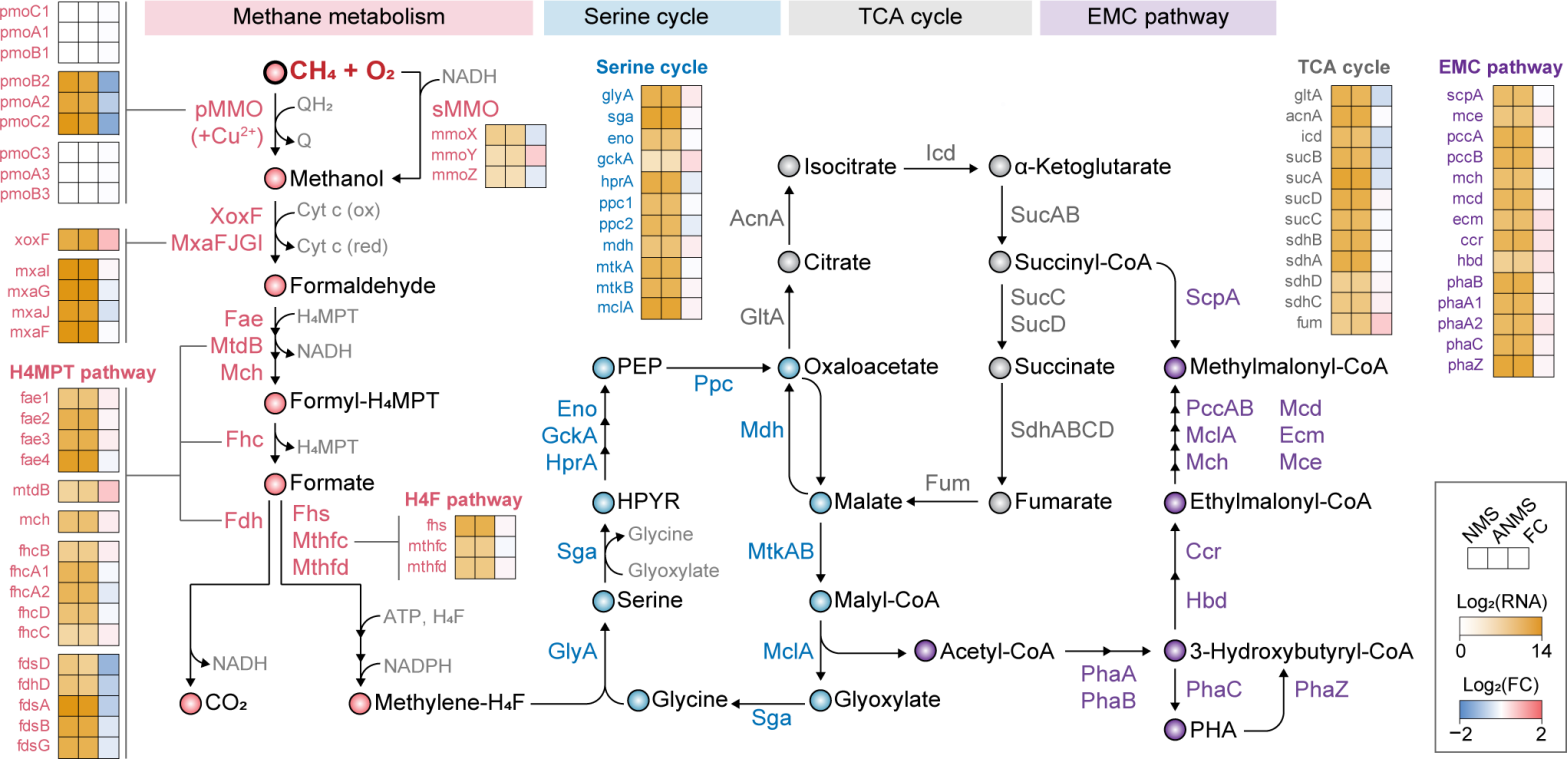
Metabolic pathways of *M. sporium* 5 with gene expression profiles. Transcriptomic changes under methane growth conditions in NMS and ANMS media are shown. Gene expression data are presented as Log_2_-transformed normalized read counts, with fold changes under ANMS conditions relative to NMS. Full transcript abundance data are available in Data S1. H4F, tetrahydrofolate; H4MPT, tetrahydromethanopterin; HPYR, hydroxypyruvate; PEP, phosphoenolpyruvate; PHA, polyhydroxyalkanoates. Corresponding locus tags for the listed gene names are provided in Data S1.

To investigate transcriptomic responses during methane utilization, we performed RNA-seq on *M. sporium* cultured in two methanotrophic media: nitrate mineral salts with or without ammonium (ANMS or NMS, respectively) (Fig. S1A and B; Table S4). Under both conditions, one of the three *pmoABC* operons exhibited more than a 37-fold increase in transcription level than *mmoXYZ* (Fig. 2; Data S1). Given that the copper concentration in both media (10 µM) exceeds the threshold required for pMMO expression (28), *M. sporium* likely primarily utilizes pMMO for methane oxidation under these conditions. Genes associated with methanol dehydrogenases, as well as the H4MPT and H4F pathways, were also highly expressed in both conditions, supporting efficient oxidation of methanol to methylene-H4F and CO_2_. Notably, *M. sporium* harbors four copies of formaldehyde-activating enzyme genes, all showing high transcript abundances, indicating robust capacity for formaldehyde detoxification and assimilation. Genes involved in the serine cycle, TCA cycle, and EMC pathway also showed high expression, suggesting active incorporation of methylene-H4F into central carbon metabolism. Furthermore, strong expression of PHA biosynthesis and depolymerization genes was observed, as previously reported in *M. trichosporium* OB3b (30).

Comparison of transcriptome profiles between two conditions revealed modest differences, with 20 upregulated and 83 downregulated genes under ANMS conditions compared to NMS (log2|fold change| > 1, adjusted *P* value < 0.05; Fig. S1C). Gene ontology (GO) enrichment analysis of downregulated genes showed enrichment of the nitrogen fixation gene cluster (*nif* operon) (Fig. S1D). This transcriptional repression reflects the typical ammonium-mediated suppression of *nif* expression to avoid energy-intensive nitrogen fixation when fixed nitrogen is readily available (31). Additionally, transcript levels of *pmoABC* genes showed at least twofold decreases under ANMS conditions, suggesting a possible inhibitory effect of ammonium on methane oxidation (Data S1). This observation aligns with previous studies reporting that ammonium inhibits MMO activity by lowering pH, generating toxic intermediates, and competing with methane due to structural similarity (32, 33). Although ammonium supplementation showed no significant phenotypic effects (Fig. S1A), transcriptomic evidence suggests that it may inhibit methane metabolism by suppressing *nif* and *pmoABC* expression in *M. sporium*. These findings indicate that ammonium-supplemented media should be carefully considered in future studies involving methane metabolism in *M. sporium*.

### Determination of consensus promoter motifs regulating methane metabolism

**Fig 3:**
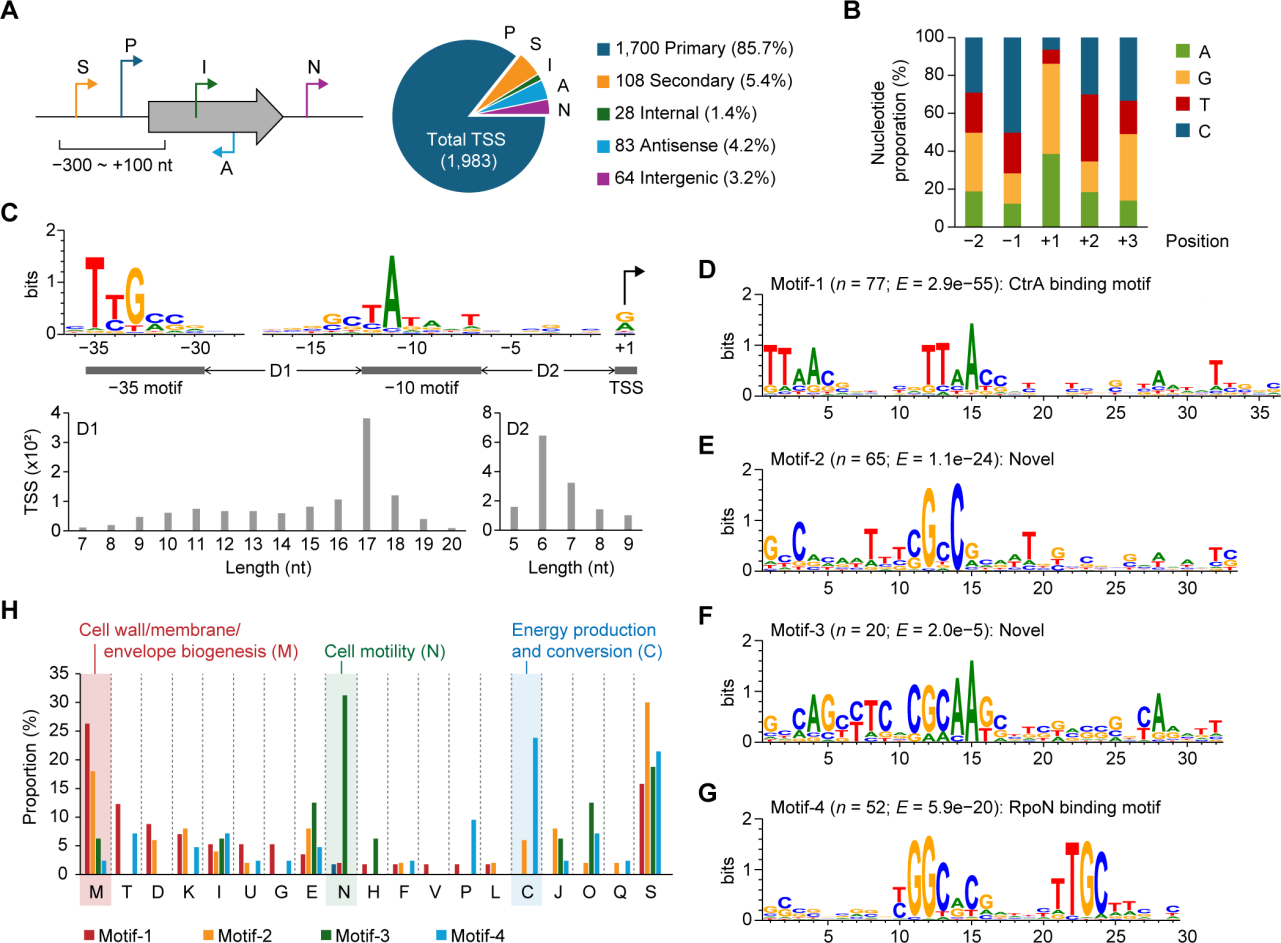
Genome-wide identification of transcription start sites and regulatory features in *M. sporium*. (**A**) Classification of identified TSSs based on their genomic positions relative to annotated genes: primary (P), secondary (S), internal (I), antisense (A), and intergenic (N) TSSs. (**B**) Nucleotide composition at TSS positions (+1) and surrounding −2 to +2 positions. At the +1 position, purines were strongly favored with over 86% (38.5% A and 47.6% G). In contrast, pyrimidines were predominant at the −1 and +2 positions with over 65% (21.5% T and 50.2% C at −1; 35.4%T and 30.1% C at +2). Despite the high genomic GC content (65%), thymine was moderately dominant at the +2 position. (**C**) Conserved promoter motifs in *M. sporium*, including −35 motif (*n* = 1,619), −10 motif (*n* = 1,371), and TSS. Each motif was found separately using MEME suite (oops mode) (38), and sequence logos were generated with WebLogo (39) using only sequences with *P* < 0.05. (**D–G**) Conserved regulatory motifs (Motifs 1–4) identified from 40 nt sequences upstream of TSSs by MEME motif search (zoops mode). The number of TSSs (*N*) and *E*-values (*E*) for each motif is shown. (**H**) Functional enrichment of genes associated with each motif, based on COG functional categories.

To identify TSSs in *M. sporium*, we employed dRNA-seq under the same conditions used for RNA-seq (Table S4). With high reproducibility (Pearson’s *r* > 0.981), we identified 1,983 TSSs, comprizing 1,496 constitutive (75.4%), 281 NMS-specific (14.2%), and 206 ANMS-specific (10.4%) sites (Table S5; Data S2). Based on their genomic locations, 1,700 TSSs (85.7%) were classified as primary, covering 39.5% of total genes (Fig. 3A). Additionally, we detected 108 secondary and 28 internal TSSs, suggesting diverse transcriptional regulation mechanisms and alternative promoters. We also identified 64 intergenic and 83 antisense TSSs, indicating the presence of putative regulatory RNAs and novel transcripts in the genome. Sequence analysis around the TSSs revealed clear nucleotide preferences (Fig. 3B). This nucleotide distribution pattern is consistent with typical bacterial TSS characteristics, where purine initiation and flanking pyrimidines enhance transcription initiation stability via base stacking interactions (34, 35). The majority of transcripts (92%) were leader mRNAs with a median 5′-UTR length of 63 nt and a conserved AG-rich RBS motif (Fig. S2A and B). In contrast, 88 leaderless transcripts were primarily associated with genes related to stress response transcriptional regulators and toxin-antitoxin systems, suggesting a role in rapid adaptation to environmental stress, as reported in other bacteria (36, 37) (Table S6; Text S1).

The *M. sporium* genome encodes 17 σ factor-related genes (Table S7). Of these, three genes encoding RpoD, RpoH, and a putative σ^70^ family σ factor were highly expressed under all conditions, suggesting their prominent roles in transcriptional regulation. To determine conserved promoter elements, 40 nt upstream sequences from identified TSSs were analyzed using the MEME motif search algorithm (38). This analysis revealed a predominant consensus motif, including highly-conserved −10 (TATAHT) and less-conserved −35 (TYGMSV) motifs, detected in 69.1% and 81.6% of promoter regions, respectively (*P* < 0.05; Fig. 3C). Spacer lengths between −35 and −10 motifs (D1), and between −10 motif and TSSs (D2), were predominantly 17 nt and 6 nt, respectively. These features are comparable to known RpoD-dependent promoter architectures in *Escherichia coli* and other α-proteobacteria (40–42), suggesting that most gene transcription in *M. sporium* is governed by the housekeeping σ factor RpoD. Corresponding consensus motifs were found in promoter regions of most genes involved in methane metabolism and central carbon metabolism illustrated in Fig. 2 (Table S8).

Among all σ factors, RpoH (σ^32^) exhibited the highest expression levels under both conditions (Table S7). In *E. coli*, σ^32^ activates genes encoding chaperones and proteases that refold and degrade misfolded proteins in response to heat shock, oxidative, and osmotic stresses (43). In *M. sporium*, genes encoding chaperones (*dnaK*, *dnaJ*, *groL*, *groES*, and *htpG*) and proteases (*ftsH*, *clpA*, *clpB*, *clpX*, and *lon*) were highly expressed (Table S9), indicating active protein quality control. In addition, reactive oxygen species (ROS)-scavenging enzymes (*sod*, *gpx*, *ccp*, and *prdx*) serving as antioxidant systems were also highly expressed, which were likely induced in response to ROS produced during methane oxidation under aerobic conditions, as previously observed in methanotrophs (44, 45). These genes are known to belong to bacterial σ^32^ regulons expressing in response to heat or oxidative stress (46–48). Although σ^32^ is known to recognize distinct −10 and −35 promoter motifs, our MEME motif search did not identify clear σ^32^ binding motifs. This is likely due to motif overlap between RpoD and RpoH target promoters, a common occurrence in α-proteobacteria that can obscure motif detection in bulk searches (49). Despite the lack of direct motif evidence, the high expression of *rpoH* and stress response-related genes strongly suggests a role for σ^32^ in controlling oxidative stress and protein homeostasis during methane metabolism in *M. sporium*. Similar RpoH-mediated stress responses have been observed in methanotrophs under oxidative stress from methanol (21) and under salt stress (24), supporting a conserved regulatory role for RpoH in stress adaptation.

### Discovery of diverse *cis*-regulatory motifs for cellular architecture and nitrogen metabolism

Analysis of 40 nt upstream sequences from TSSs revealed several conserved motifs (Motifs 1–4), in addition to the housekeeping RpoD binding motif (Motif 5) (Fig. 3D through G; Data S3). Functional classification of the genes associated with these motifs using Clusters of Orthologous Groups (COG) categories showed distinct enrichment patterns (Fig. 3H). Motifs 1 and 2 were predominantly associated with genes involved in cell wall/membrane/envelope biogenesis (COG category M), Motif 3 with cell motility (N), and Motif 4 with energy production and conversion (C). These patterns suggest that the motifs are recognized by distinct transcription factors or σ factors that regulate specific functional gene sets.

Motif 1 features a well-conserved TTAA-N_7_-TTAA motif (Fig. 3D; Data S3), which matches the binding motif of the CtrA transcription regulator conserved in α-proteobacteria (50). CtrA is known to regulate genes involved in cell cycle progression, division, envelope biogenesis, and outer membrane assembly (50, 51). In *M. sporium*, a CtrA homolog (MSP_003086) sharing 83.6% identity with *Caulobacter crescentus* was highly expressed (98th percentile RPKM) under all conditions. The enrichment of Motif 1 in promoters of genes related to COG categories M (cell wall/membrane/envelope biogenesis) and D (cell cycle control, cell division, and chromosome partitioning) further supports the role of CtrA in regulating cellular architecture and division in *M. sporium* (Fig. 3H).

Motifs 2 and 3 appear to be novel, as they did not match any known motifs in public databases (Fig. 3E and F; Data S3). Notably, genes associated with Motif 3 were primarily downregulated under ANMS conditions, whereas Motifs 1 and 2 were linked to genes with consistent expression across conditions. Motif 3 was enriched in the promoter regions of flagellar genes (*flgB*, *motA*, *flgF*, *fliF*, and *flgE*) located in the *flg* gene cluster (MSP_001697-001750), which encodes key components of the flagellar basal body and hook. This aligns with the enrichment of the cell motility category (COG N), suggesting a potential regulatory role of Motif 3 in flagellar biogenesis.

Motif 4 contained a conserved RpoN (σ^54^) binding motif (YGGCR-N_6_-TTGC), consistent with known −12 and −24 elements recognized by σ^54^, which requires bacterial enhancer-binding proteins (bEBPs or σ^54^ activators) for transcription initiation (52) (Fig. 3G; Data S3). RpoN-associated promoters were identified upstream of nitrogen metabolism genes, including *nif* operon (MSP_003382-003414) and nitrate transporters (*nrtABC*, *nirAB*; MSP_002299-002305). These genes were transcriptionally downregulated approximately twofold under ANMS conditions (Data S1, S3). The presence of *nifA* (MSP_003382), encoding a known σ^54^ activator within the *nif* operon, supports RpoN-dependent regulation of nitrogen metabolism in *M. sporium*, in response to ammonium supplementation.

### Genome-wide identification and classification of transcript 3′-end positions

**Fig 4:**
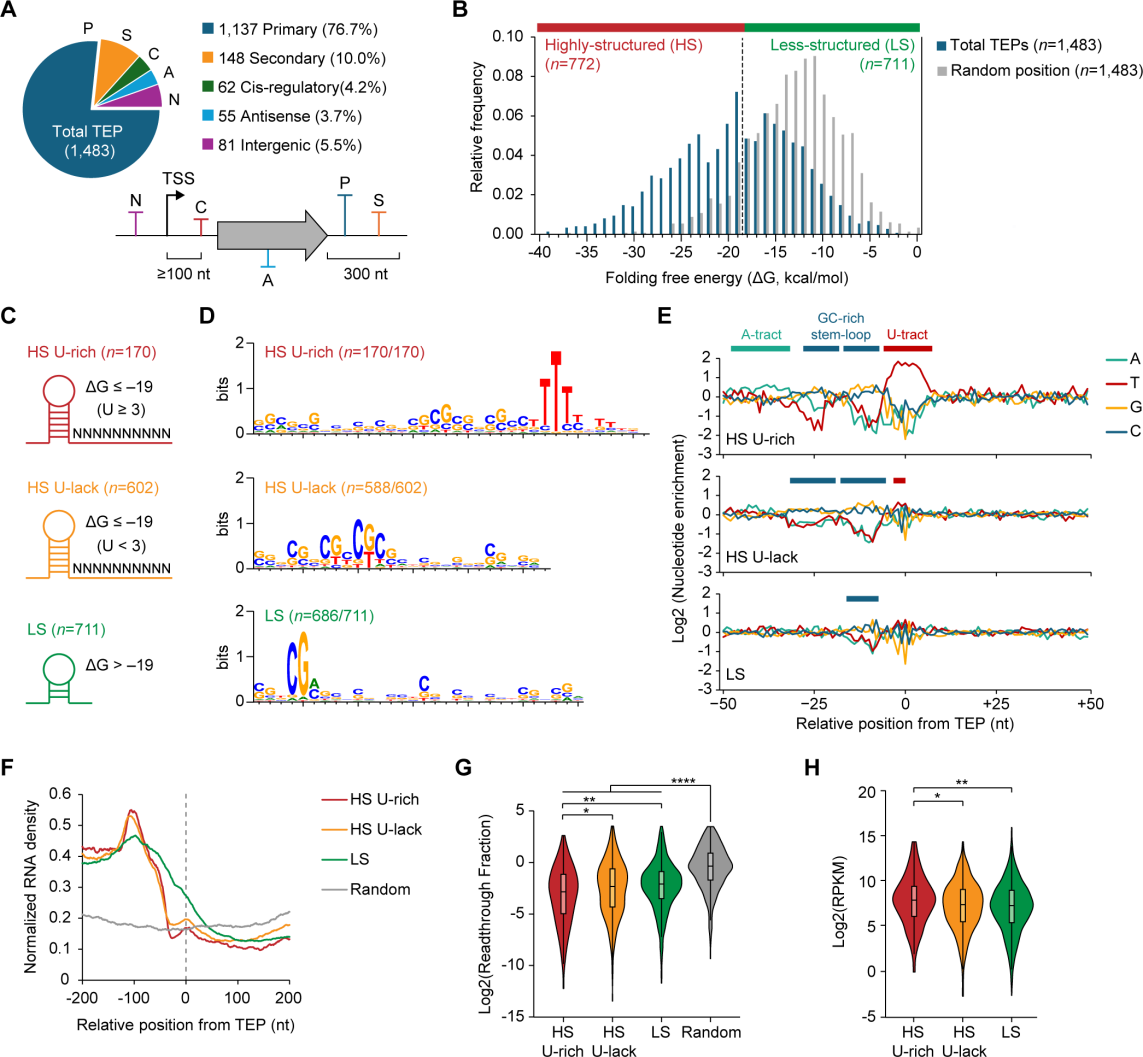
Classification and characterization of three classes of TEPs in *M. sporium*. (**A**) Classification of TEPs based on their genomic positions relative to annotated genes: primary (P), secondary (S), *cis*-regulatory (C), antisense (A), and intergenic (N) TEPs. (**B**) Distribution of RNA folding free energy (Δ*G*) values calculated from 40 nt sequences upstream of TEPs and randomly selected intergenic positions (*n* = 1,483). TEPs were classified as highly structured (HS) and less structured (LS) TEPs based on a Δ*G* threshold of −19 kcal/mol, corresponding to the median of the total TEP distribution. (**C**) Schematic representation of the three TEP groups classified based on Δ*G* values and the number of uridines downstream of predicted stem-loop structures. (**D**) Conserved sequence motifs detected by MEME algorithms for each TEP group. Motif detection was performed using sequences spanning 41 nt upstream to 20 nt downstream of each TEP. Only sequences with *P* < 0.05 were used for motif generation. (**E**) Nucleotide enrichment analysis from −50 to +50 nt relative to TEPs. (**F**) RNA-seq read density around TEPs under ANMS conditions. Random represents 1,483 randomly selected intergenic positions. (**G**) Readthrough fraction for each TEP group, calculated as the ratio of average normalized RNA read counts between the upstream (−200 to 0 nt) and downstream (0 to +200  nt) regions of TEPs or random positions. RNA-seq data from ANMS conditions were used. Outliers above the 95th percentile were excluded. (**H**) Gene expression levels associated with each TEP group. RPKM values were calculated for each biological replicate under ANMS conditions, and the average values were used. *, *P*  <  0.05; **, *P*  <  0.01; ***, *P*  <  0.001; and ****, *P*  <  0.0001 (Mann-Whitney *U*-test, two-sided).

To determine TU architecture, TEP information is required to define transcript boundaries. To this end, we performed Term-seq, which yielded high reproducible results (Pearson’s *r* > 0.984; Table S4). This analysis identified 1,483 TEPs, comprizing 806 shared (54.3%), 420 NMS-specific (28.3%), and 257 ANMS-specific (17.3%) TEPs (Table S5; Data S2). Classification of TEPs based on genomic positions revealed 1,137 primary TEPs (76.7%) located within 300 nt downstream of gene 3′-ends, accounting for 26.7% of total genes (Fig. 4A; Data S2). Additionally, we identified 148 secondary, 81 intergenic, 55 antisense TEPs, and 62 *cis*-regulatory TEPs located within 5′-UTRs. These findings suggest diverse mechanisms for transcription termination and the presence of regulatory RNAs in the *M. sporium* genome.

Analysis of 3′-UTR lengths between primary TEPs and gene 3′-ends showed a median length of 89 nt, with the most frequent range between 50 and 59 nt (Fig. S3A). Rare occurrence of short 3′-UTRs < 20 nt (3.2%) suggested predominant 3′-UTR-mediated regulation in *M. sporium*. We calculated the RNA folding free energy (Δ*G*) of 40 nt upstream sequences from TEPs and random intergenic positions. The Δ*G* values for TEPs (median Δ*G*: −19.3 kcal/mol) were significantly lower than those for random positions (median Δ*G*: −13.2 kcal/mol), suggesting the potential for stem-loop structure formation upstream of TEPs (Fig. 4B). Based on Δ*G* distribution, approximately half of the TEPs overlapped with the Δ*G* values of random intergenic positions, suggesting relatively low structural stability. Accordingly, we divided TEPs into two groups: highly structured (HS; *n* = 772) with Δ*G* ≤ –19 kcal/mol and less structured (LS; *n* = 711) with Δ*G* > –19 kcal/mol. To further classify HS TEPs by terminator shape, we analyzed U content within 10 nt downstream of predicted stem-loops structures, based on previously defined L- and I-shaped terminators (53) (Fig. S3B). We defined HS U-rich TEPs (*n* = 170) as those containing ≥3 Us following the stem-loop, with the remainder classified as HS U-lack TEPs (*n* = 602) (Fig. 4C). Motif analysis of the three TEP classes revealed a broadly conserved GC-rich region across all groups, whereas a distinct downstream U-tract was exclusively observed in HS U-rich TEPs, consistent with canonical Rho-independent intrinsic terminator features (Fig. 4D).

### Characterization of transcript 3′-end sequences for transcription termination

To investigate regulatory elements involved in transcription termination, we analyzed nucleotide enrichment across regions spanning −50 to +50 nt relative to TEPs. In HS U-rich TEPs, we observed sequence features characteristic of canonical L-shaped intrinsic terminators: GC-rich stem-loop regions (−30 to −6), downstream U-rich tracts (−5 to +5), and upstream A-tracts (−45 to −32), comparable to intrinsic terminators in *E. coli* (54) (Fig. 4E). In contrast, HS U-lack TEPs lacked A-tracts, showed weaker GC-rich stem-loops, and had substantially reduced U content around TEPs. These features are consistent with the I-shaped intrinsic terminators commonly found in GC-rich bacterial genomes (53). Notably, I-shaped terminators accounted for 78% of HS TEPs in *M. sporium*, compared to 22% for L-shaped terminators, reflecting a preference that aligns with previous observations in other high-GC bacteria.

Unlike HS TEPs, LS TEPs exhibited only weak enrichment of GC-rich regions and lacked distinct sequence motifs (Fig. 4D and E). Nevertheless, base-pairing interaction analysis of the 100 nt upstream regions of LS TEPs showed weak RNA secondary structure formation, albeit with lower stability than HS TEPs (Fig. S3C). These TEPs are likely derived from alternative mechanisms such as post-transcriptional RNA processing (55) or Rho-dependent transcription termination (54), given the presence of the Rho factor gene (MSP_002761) in the *M. sporium* genome, which is expressed under both tested conditions.

To assess termination patterns and strengths, we analyzed RNA abundance around TEPs. All three TEP classes showed a clear decrease in RNA levels upstream of TEPs (−100 to −1), supporting their functional roles in transcription termination (Fig. 4F). Notably, the two HS TEP classes showed sharper declines in RNA abundance near TEPs and significantly lower readthrough levels than LS TEPs, which exhibited more gradual decreases (Fig. 4G; Fig. S3D). These differences are supported by structural features, as HS TEPs possessed longer stem and shorter loop lengths than LS TEPs (Fig. S3E), suggesting more stable stem-loop formation and higher termination efficiency. This observation aligns with findings in *E. coli*, where longer stems and shorter loop structures contribute to more effective intrinsic termination (56). Strong intrinsic terminators are particularly important for preventing leaky readthrough in highly expressed genes. Consistent with this, both HS TEP classes were associated with significantly higher gene expression levels than LS TEPs (Fig. 4H; Fig. S3F). Particularly, HS U-rich TEPs were most frequently associated with the highest expression levels, reinforcing their role as the most robust terminators in *M. sporium*.

Taken together, *M. sporium* primarily employs I-shaped intrinsic terminators for transcription termination, while L-shaped terminators are preferentially used to terminate highly expressed genes and prevent unwanted transcriptional readthrough. Although LS TEPs exhibit weaker termination signals, they are still functional and may result from RNA processing or Rho-dependent termination mechanisms.

### Determination of TUs by integrating TSSs and TEPs

**Fig 5:**
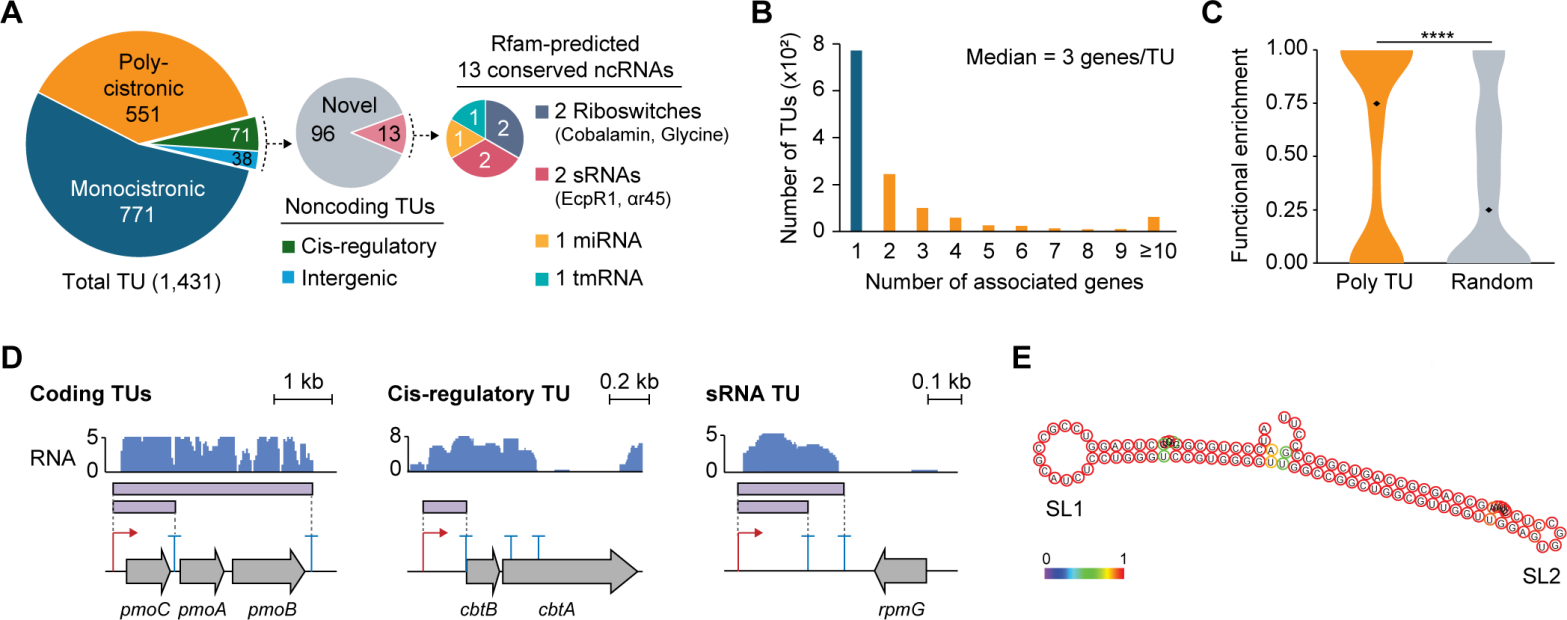
Determination of transcription units (TUs) in *M. sporium*. (**A**) Categorization of detected TUs and functional prediction of non-coding TUs based on ncRNA families predicted using the Rfam database (59). (**B**) Distribution of the number of genes per coding TUs. Monocistronic and polycistronic TUs are indicated in blue and orange, respectively. (**C**) Functional relatedness of genes within the same polycistronic (Poly) TU compared to randomly selected genes (Random). The functional enrichment score was defined as the maximum ratio of genes assigned to a single COG category to the total number of COG categories per TU. Genes without any assigned COG category were excluded. Dots represent the median values, and statistical significance was determined based on *P* values (****, *P*  <  0.0001; Mann-Whitney *U*-test, two-sided). (**D**) Examples of identified TU types. RNA-seq profiles are shown as Log_2_-transformed RNA abundance levels. (**E**) Predicted secondary structure of the putative sRNA EcpR1 using RNAfold (60). Nucleotides are colored according to base-pairing probabilities.

Integration of TSSs and TEPs determined 1,431 TUs, comprizing 771 monocistronic, 551 polycistronic, 71 *cis*-regulatory, and 38 intergenic TUs (Data S2; Fig. 5A). Polycistronic TUs had a median of 3.0 genes, comparable to those in *E. coli* (3.4 genes) (57) and *B. subtilis* (3.7 genes) (58) (Fig. 5B). COG enrichment analysis of genes within polycistronic TUs showed significantly higher functional relatedness compared to randomly selected neighboring gene sets (*P* < 6.7 × 10^–15^) (Fig. 5C). This indicates that functionally related genes, such as *pmoABC* operon, are frequently co-transcribed within the same polycistronic TU, allowing coordinated regulation of genes involved in shared metabolic processes (Fig. 5D).

Analysis of non-coding TUs (*cis*-regulatory and intergenic) using the Rfam database (59) revealed six conserved non-coding RNA (ncRNA) families (Table S10; Fig. 5A). For *cis*-regulatory elements, riboswitches for cobalamin and glycine were identified in the 5′-UTRs of genes involved in cobalamin biosynthesis and glycine cleavage system, consistent with their conserved genomic context across bacteria (61) (Fig. 5D). Notably, two previously unannotated sRNAs, EcpR1 and αr45, were identified in intergenic regions (Fig. 5D). These sRNAs are conserved among α-proteobacteria (62, 63). While the functional role of αr45 remains unclear, EcpR1 is a well-characterized stress-induced sRNA that modulates cell cycle progression in *Sinorhizobium meliloti* by repressing the expression of cell cycle regulators such as *dnaA* and *gcrA* at the post-transcriptional level through base-pairing via its conserved GC-rich stem-loop (SL1) (62, 63). In *M. sporium*, the putative EcpR1 was located adjacent to *rpmG*, in the same orientation as in *S. meliloti* (62), and its predicted secondary structure formed two stem-loop domains, SL1 and SL2, consistent with *S. meliloti* EcpR1 (Fig. 5E). Given the activation of stress response genes and discovery of a CtrA-regulated motif associated with cell cycle control during growth on methane (Table S9), it is likely that EcpR1 in *M. sporium* also contributes to the regulation of stress responses and cell division, functioning as a stress-induced sRNA, similar to *S. meliloti* EcpR1.

## DISCUSSION

In this study, we integrated genome sequencing, RNA-seq, dRNA-seq, and Term-seq to elucidate the transcriptional regulatory landscape of *M. sporium* 5. We identified 1,983 TSSs and 1,483 TEPs, which collectively defined 1,431 TUs. These data enabled genome-scale identification of genetic regulatory elements, including promoters, UTRs, terminators, and regulatory RNAs, providing the first comprehensive view of transcriptional regulation in a methanotroph.

Comparative genomic analysis confirmed key features of type II methanotrophs in *M. sporium*. Transcriptome profiling under NMS and ANMS conditions revealed two key responses: (i) high expression of methane metabolism genes, and (ii) ammonium-induced transcriptional repression of the *nif* operon along with a twofold reduction in *pmoABC* expression, suggesting a potential inhibitory effect on both nitrogen fixation and methane oxidation. TSS analysis revealed widespread RpoD-dependent promoter motifs upstream of genes involved in methane and central carbon metabolism, while expression patterns suggested a potential regulatory role for RpoH in stress response. In addition, three distinct classes of TEPs were identified, each with unique sequence features and termination strengths.

Beyond conserved σ factor-mediated regulation, we discovered multiple *cis*-regulatory promoter motifs associated with genes involved in nitrogen metabolism, cell cycle control, cell envelope biogenesis, cell division, and motility. Notably, a conserved CtrA-binding motif was detected upstream of genes related to cell cycle control and cell division. While further investigation is required to determine the σ factors or transcriptional regulators acting on these motifs and to fully elucidate their regulatory roles, systematically mapped TSSs or TEPs across the genome provide a foundational resource for metabolic engineering of methanotrophs. The regulatory elements identified in this study can serve as a library of genetic parts for fine-tuning gene expression, enabling improvements in methane bioconversion efficiency and the development of optimized pathways for producing value-added biochemicals from methane.

We also discovered EcpR1, a stress-responsive sRNA known to modulate cell cycle progression in *S. meliloti*. The conservation of its sequence, secondary structure, and genomic context in *M. sporium*, along with the presence of CtrA motifs, suggests a potential stress-responsive regulatory network linking environmental signals to cell division. To further explore its potential function, we performed target mRNA prediction using IntaRNA (64) and sRNARFTarget (65). However, the predicted targets showed minimal overlap and lacked consistent enrichment of regulatory motifs or functional categories (e.g., cell cycle or stress response). This lack of concordance reflects the limitations of computational predictions alone, particularly without experimental validation, and aligns with previous reports of high false-positive rates and poor consistency between tools (66). Therefore, future experimental approaches, such as gene expression profiling, immunoprecipitation, and pull-down assays (66), will be essential to elucidate the true functional targets and regulatory roles of EcpR1 in *M. sporium*.

In conclusion, this study represents the first application of high-throughput sequencing approaches to comprehensively define the transcriptional regulatory landscape of a methanotroph. Genome-wide identification of TSSs, TEPs, and TUs has expanded our understanding of gene regulation associated with diverse cellular responses including methane metabolism in *M. sporium*. The discovery of novel *cis*-regulatory motifs and sRNAs underscores the complexity of transcriptional regulation in methanotrophs and highlights the need for future studies to functionally characterize these elements and their regulatory networks.

## MATERIALS AND METHODS

### Bacterial strains and culture conditions

*M. sporium* 5 (DSM 17706) was obtained from the Leibniz Institute DSMZ-German Collection of Microorganisms and Cell Cultures (DSMZ, Germany). Nitrate mineral salt (NMS) and ammonium nitrate mineral salt (ANMS) medium (pH 6.8) were used to cultivate *M. sporium* 5, as previously described (67, 68). Briefly, the culture was prepared in 1 L Duran bottles containing 200 mL NMS or ANMS medium with 10 µM CuCl_2_. After replacing 50% of the headspace with methane (99.95%), cultures were incubated at 30°C and 200 rpm. For multi-omics analyzes, the cells were harvested at mid-exponential phase (OD_600_ = 0.35).

### Genome-seq, RNA-seq, dRNA-seq, and Term-seq

Genomic DNA was extracted using the Wizard Genomic DNA Purification Kit (Promega). The complete genome sequence of *M. sporium* 5 was obtained using PacBio Sequel II and Illumina NovaSeq 6000 (2 × 150 bp) platforms. *De novo* assembly was performed using Flye (v2.8.3) (69), followed by circularization with Circlator (v1.5.5) (70), and genome annotation using NCBI PGAP (71). Total RNA was extracted using the RNAsnap protocol (72), followed by rRNA depletion with the RiboRid method (73) using custom anti-rRNA probes designed to target rRNAs of *M. sporium* 5. RNA-seq libraries were prepared using the TruSeq Stranded mRNA Library Prep Kit (Illumina), while dRNA-seq and Term-seq libraries were constructed following published protocols with minor modifications (16, 34, 73). Library quality was validated using Qubit 4 fluorometer (Invitrogen) and TapeStation 4150 (Agilent). RNA-seq libraries were sequenced on Illumina MiSeq (2 × 75 bp), while dRNA-seq and Term-seq libraries were sequenced on Illumina NextSeq 1000 (1 × 100 bp). All sequencing reads were processed and mapped to the *M. sporium* genome using CLC Genomics Workbench 6.5.1 (Qiagen). Primers used are listed in Table S11. Additional details on sequencing library preparation and data processing are provided in Methods S1 in Supplementary information.

### Genome and transcriptome analysis

Complete genome sequences of 12 methanotrophs (Table S2) were retrieved from NCBI. Comparative genomic and genome-based phylogeny analyzes were performed using Orthofinder (27). Protein sequences were compared using BLASTP (*E*-value < 1e−3). For RNA-seq analysis, uniquely mapped reads were retrieved and normalized using Bioconductor package DEseq2 with default parameters (74). Functional enrichment analysis was conducted using ClusterProfiler (75), based on KEGG Orthology (blastKOALA [76]) or GO (EggNOG [77]) annotations. A Benjamini–Hochbergcorrected *P* < 0.05 was considered significant.

### Identification of TSSs and TEPs

TSSs and TEPs were identified from uniquely mapped dRNA-seq reads and Term-seq reads, respectively, using modified versions of previously described methods (34, 78, 79). All identified TSSs and TEPs were manually curated with RNA-seq profiles and classified into five categories based on their genomic positions relative to adjacent genes. Results from two conditional libraries were merged within ±5 nt to determine total TSSs and TEPs (Data S2). The folding free energy (Δ*G*) of RNA secondary structures was calculated for 40 nt upstream sequences using RNAfold at 30°C (60). Details are available in Methods S1.

### Motif detection

Consensus promoter motifs (40 nt upstream of TSSs) and terminator motifs (41 nt upstream to 20 nt downstream of TEPs) were identified using MEME with oops mode (38). Diverse *cis*-regulatory motifs were identified using zoops mode. Only sequences with *P* < 0.05 were used to generate motifs using Weblogo (39).

### Determination of TUs

TUs were determined as previously described (79) by integrating TSS, TEP, and RNA-seq data, defining each TU as the region between a TSS and a TEP. TUs identified in both conditions were merged within a ± 5 nt range to make a total TU list (Data S2). TUs were categorized into coding and non-coding TUs. Functional annotation of non-coding TUs was performed using the Rfam database, with an *E*-value threshold of 1e−5, and only the top hits were retrieved (59).

## Data Availability

The complete genome sequence of *M. sporium* 5 has been deposited in GenBank under the accession numbers CP189553–CP189555. All sequencing data (RNA-seq, dRNA-seq, and Term-seq) are available in the SRA under the BioProject accession number PRJNA1238525.

## SUPPLEMENTAL MATERIAL

Data S1 spectrum.01281-25-s0001.xlsx Transcriptome data.

Data S2 spectrum.01281-25-s0002.xlsx List of total TSSs, TEPs, and TUs identified in *M. sporium* 5.

Data S3 spectrum.01281-25-s0003.xlsx Promoter motifs identified in *M. sporium* 5.

Supplemental figures, tables, methods, and text spectrum.01281-25-s0004.pdf Fig. S1 to S3, Tables S1 to S11, Methods S1, and Text S1.

ASM does not own the copyrights to Supplemental Material that may be linked to, or accessed through, an article. The authors have granted ASM a non-exclusive, world-wide license to publish the Supplemental Material files. Please contact the corresponding author directly for reuse.
