# Supplementary material for: Determination of the transcription unit landscape and associated regulatory elements in Methylosinus sporium 5
Source: Microbiol Spectr. 2025 Aug 6;13(9):e01281-25. doi: 10.1128/spectrum.01281-25 (PMC12403871; doi:10.1128/spectrum.01281-25)
Supplement: Supplemental figures, tables, methods, and text — Fig. S1 to S3, Tables S1 to S11, Methods S1, and Text S1. [file spectrum.01281-25-s0004.pdf]

Supplementary Information

Determination of the transcription unit landscape and associated regulatory elements in *Methylosinus sporium* 5

Jiyun Bae[a], Dong-Uk Song[b,c], Hyewon Lee[c,d], Seung-Goo Lee[b,c,d], and Byung-Kwan Cho[a,b,e]*

[a] Department of Biological Sciences, Korea Advanced Institute of Science and Technology, Daejeon 34141, Republic of Korea

[b] Graduate School of Engineering Biology, Korea Advanced Institute of Science and Technology, Daejeon 34141, Republic of Korea

[c] Synthetic Biology Research Center and the K-Biofoundry, Korea Research Institute of Bioscience and Biotechnology (KRIBB), Daejeon 34141, Republic of Korea

[d] Department of Biosystems and Bioengineering, KRIBB School of Biotechnology, University of Science & Technology, Daejeon 34113, Republic of Korea

[e] KI for the BioCentury, Korea Advanced Institute of Science and Technology, Daejeon 34141, Republic of Korea

*Corresponding author: Byung-Kwan Cho (bcho@kaist.ac.kr)

CONTENTS

Supplementary Tables

Table S1. General features of the *Methylosinus sporium* 5 genome

Table S2. List of methanotrophs with complete genome sequences

Table S3. Pan-genome analysis of methanotrophs

Table S4. Sequencing statistics for RNA-seq, dRNA-seq, and Term-seq

Table S5. Statistics of identified transcription start sites and transcript 3′-end positions

Table S6. Expression level of sigma factor-related genes

Table S7. Consensus promoter motifs detected in genes involved in methane metabolism

Table S8. Stress response related genes highly expressed at 90th percentile RPKM levels

Table S9. Leaderless mRNAs associated genes

Table S10. Predicted ncRNAs from noncoding TUs using Rfam database

Table S11. List of oligonucleotides used in this study

Supplementary Figures

Figure S1. RNA-seq analysis of *M. sporium* 5

Figure S2. Determination of 5′-UTRs in *M. sporium* 5

Figure S3. Analysis of three classes of TEPs identified in *M. sporium*

Supplementary Text

Text S1. Determination of 5′-untranslated regions

Supplementary Methods

Method S1. Sequencing and data analysis

Other Supplementary Data (Separate files)

Data S1. Transcriptome data

Data S2. List of total TSSs, TEPs, and TUs identified in *M. sporium* 5

Data S3. Promoter motifs identified in *M. sporium* 5

Table S1. General features of the *Methylosinus sporium* 5 genome

Feature	Chromosome	Plasmid 1	Plasmid 2	Total
Size (bp)	4,148,301	270,036	115,598	4,533,935
GC content (%)	64.89	63.93	62.41	64.77
Total genes	3,966	173	97	4,236
Coding sequences (CDSs)	3,906	173	97	4,176
- Protein coding sequences	3,850	165	83	4,098
- Pseudo genes (CDS without protein)	56	8	14	78
RNA coding genes	60	0	0	60
- rRNAs (5S, 16S, 23S)	6 (2, 2, 2)	0	0	6
- tRNAs	50	0	0	50
- ncRNAs	3	0	0	3
- tmRNAs	1	0	0	1

Table S2. List of methanotrophs with complete genome sequences

Organism	NCBI RefSeq assembly	Chromosome size (bp)	GC %	Plasmid count
Methylomonas sp. DH-1	GCF_001644685.1	4,849,532	56.5	1
Methylobacter sp. YRD-M1	GCF_026727675.1	4,653,847	51.5	2
Methylotuvimicrobium alcaliphilum 20Z	GCF_000968535.2	4,668,296	48.5	1
Methylotuvimicrobium buryatense 5GB1C	GCF_005931095.1	4,998,879	48.5	-
Methylococcus capsulatus str. Bath	GCF_000008325.1	3,304,561	63.5	-
Methylosinus trichosporium OB3b	GCF_002752655.1	4,508,832	66.0	3
Methylosinus sp. C49	GCF_009936375.1	3,918,175	65.0	4
Methylosinus sporium 5	This study	4,148,301	64.9	2
Methylocystis bryophila DSM 21852	GCF_027925445.1	4,540,773	63.0	1
Methylocystis heyeri H2	GCF_004802635.2	4,551,947	63.0	2
Methylocystis parvus OBBP	GCF_027571405.1	4,076,007	63.5	2
Methylocystis sp. SC2	GCF_000304315.1	3,773,444	63.5	-

Table S3. Pan-genome analysis of methanotrophs

Species	Core	Type-specific	Species-specific	Singleton
Methylomonas sp. DH-1	935	313	45	693
Methylobacter sp. YRD-M1			50	698
Methylotuvimicrobium alcaliphilum 20Z			8	195
Methylotuvimicrobium buryatense 5GB1C			9	225
Methylococcus capsulatus str. Bath			18	405
Methylosinus trichosporium OB3b		460	23	513
Methylosinus sp. C49			6	242
Methylosinus sporium 5			5	271
Methylocystis bryophila DSM 21852			12	446
Methylocystis heyeri H2			16	481
Methylocystis parvus OBBP			24	435
Methylocystis sp. SC2			14	333

Table S4. Sequencing statistics for RNA-seq, dRNA-seq, and Term-seq

Sample name	Raw data		After trimming			Mapping (reference genome: *M. sporium* 5)			
	Total reads	Mean length	Trimmed reads	%	Mean length	Mapped reads	%	Mean length	Coverage
RNA-seq (MiSeq, 2x75 bp)									
ANMS_1	7,476,764	75.5	7,457,976	99.8%	74.4	7,065,866	94.9%	74.7	116.3
ANMS_2	8,509,092	75.5	8,477,122	99.6%	73.7	7,834,748	92.7%	74.3	128.3
ANMS_3	9,141,910	75.5	9,123,369	99.8%	74.5	8,734,108	95.9%	74.7	143.9
NMS_1	6,894,290	75.5	6,869,801	99.6%	73.9	6,411,955	93.6%	74.4	105.2
NMS_2	7,767,192	75.5	7,737,824	99.6%	73.7	7,218,711	93.6%	74.3	118.3
NMS_3	8,576,400	75.5	8,547,642	99.7%	73.9	7,984,529	93.7%	74.4	131.0
TSS-seq (NextSeq 1000, 1x100 bp)									
ANMS_TEX_1	6,389,274	79.1	3,969,884	62.1%	89.4	2,186,018	55.1%	97.2	46.8
ANMS_TEX_2	5,249,387	86.4	3,886,001	74.0%	93.2	2,327,406	59.9%	98.5	50.6
ANMS_TEX_3	4,599,027	88.4	3,797,658	82.6%	95.1	2,688,516	70.8%	97.7	57.9
NMS_TEX_1	3,815,800	94.2	3,528,994	92.5%	96.7	3,071,832	87.1%	98.3	66.6
NMS_TEX_2	4,793,362	91.0	4,025,366	84.0%	95.7	2,853,598	70.9%	98.6	62.0
NMS_TEX_3	4,857,678	86.0	3,822,721	78.7%	93.3	2,790,120	73.0%	96.2	59.2
ANMS_1	6,406,958	88.9	5,388,092	84.1%	93.9	3,064,660	56.9%	97.6	66.0
ANMS_2	6,218,978	86.5	5,388,092	84.1%	93.9	3,129,336	61.0%	96.6	66.7
ANMS_3	5,529,321	90.6	4,950,322	89.5%	94.0	3,422,865	69.1%	96.4	72.7
NMS_1	6,070,761	93.1	5,677,103	93.5%	95.3	4,845,451	85.4%	96.4	103.0
NMS_2	5,921,951	92.8	5,513,776	93.1%	94.9	4,435,983	80.5%	96.2	94.1
NMS_3	4,617,812	94.4	4,299,642	93.1%	96.7	3,313,726	77.1%	98.0	71.7
Term-seq (NextSeq 1000, 1x100 bp)									
Term_ANMS_1	4,649,076	86.3	3,762,351	80.9%	93.5	3,420,586	90.9%	95.2	71.8
Term_ANMS_2	3,982,734	90.3	3,425,308	86.0%	94.7	3,132,717	91.5%	95.6	66.1
Term_ANMS_3	3,818,935	90.2	3,280,104	85.9%	94.7	3,007,439	91.7%	95.7	63.5
Term_NMS_1	6,081,136	84.3	4,710,476	77.5%	93.7	4,367,125	92.7%	95.2	91.7
Term_NMS_2	4,699,230	87.7	3,871,334	82.4%	94.3	3,545,535	91.6%	95.4	74.6
Term_NMS_3	5,410,035	88.4	4,506,783	83.3%	94.4	4,110,629	91.2%	95.5	86.6

Table S5. Statistics of identified transcription start sites and transcript 3′-end positions

**Transcription start sites (TSS)**	**Total TSS in each condition (n = 1,777; 1,702)**					**Shared TSS (n = 1,496)**			**Condition-specific TSS (n = 281; 206)**					**Total TSS (n = 1,983)**		
		**NMS**		**ANMS**		**NMS & ANMS**				**NMS**		**ANMS**		**NMS + ANMS**		
**Category**		**#**	**%**	**#**	**%**		**#**	**%**		**#**	**%**	**#**	**%**	**#**	**%**	
Primary (P)	P	1531	86.2	1461	85.8	P	1265	84.6	P	240	85.4	169	82.0	1700	85.7	a
Secondary (S)	S	81	4.6	75	4.4	S	22	1.5	S	32	11.4	27	13.1	108	5.4	b
Internal (I)	I	28	1.6	28	1.6	I	28	1.9	I	0	0.0	0	0.0	28	1.4	
Antisense (A)	A	76	4.3	76	4.5	A	69	4.6	A	7	2.5	7	3.4	83	4.2	
Intergenic (N)	N	61	3.4	62	3.6	N	59	3.9	N	2	0.7	3	1.5	64	3.2	
P in NMS and S in ANMS	-	-	-	-	-	P/S	26	1.7	-	-	-	-	-	-	-	
S in NMS and P in ANMS	-	-	-	-	-	S/P	27	1.8	-	-	-	-	-	-	-	
**Total**		**1777**	100	**1702**	100		**1496**	100		**281**	100	**206**	100	**1983**	100	

**Transcript 3′-end positions (TEP)**	**Total TEP in each condition (n = 1,226; 1,063)**					**Shared TEP (n = 806)**			**Condition-specific TEP (n = 420; 257)**					**Total TEP (n = 1,483)**		
		**NMS**		**ANMS**		**NMS & ANMS**				**NMS**		**ANMS**		**NMS + ANMS**		
**Category**		**#**	**%**	**#**	**%**		**#**	**%**		**#**	**%**	**#**	**%**	**#**	**%**	
Primary (P)	P	951	77.6	843	79.3	P	629	78.0	P	299	71.2	186	72.4	1137	76.7	c
Secondary (S)	S	121	9.9	79	7.4	S	31	3.8	S	66	15.7	27	10.5	148	10.0	b
Cis-regulatory (C)	C	56	4.6	43	4.0	C	35	4.3	C	17	4.0	6	2.3	62	4.2	d
Antisense (A)	A	32	2.6	37	3.5	A	14	1.7	A	18	4.3	23	8.9	55	3.7	
Intergenic (N)	N	66	5.4	61	5.7	N	46	5.7	N	20	4.8	15	5.8	81	5.5	
P in NMS and S in ANMS	-	-	-	-	-	P/S	21	2.6	-	-	-	-	-	-	-	
P in NMS and C in ANMS	-	-	-	-	-	P/C	2	0.2	-	-	-	-	-	-	-	
S in NMS and P in ANMS	-	-	-	-	-	S/P	24	3.0	-	-	-	-	-	-	-	
C in NMS and P in ANMS	-	-	-	-	-	C/P	4	0.5	-	-	-	-	-	-	-	
**Total**		**1226**	100	**1063**	100		**806**	100		**420**	100	**257**	100	**1483**	100	

a, P(NMS,ANMS)+P/S

b, S(NMS,ANMS)+S/P

c, P(NMS,ANMS)+P/S+P/C

d, C(NMS,ANMS)+C/P

Table S6. Leaderless mRNAs associated genes

TSS position	Locus tag	Annotation
18186	MSP_000011	hypothetical protein
183304	MSP_000150	fluoride efflux transporter CrcB
207230	MSP_000175	YgiQ family radical SAM protein
228007	MSP_000200	exodeoxyribonuclease VII large subunit
263256	MSP_000230	IS66 family transposase
264713	MSP_000234	hypothetical protein
272958	MSP_000244	Fe2+-dependent dioxygenase
298258	MSP_000269	Co2+/Mg2+ efflux protein ApaG
396669	MSP_000401	DUF459 domain-containing protein
551810	MSP_000550	GSCFA domain-containing protein
562031	MSP_000557	ATP-binding cassette domain-containing protein
595914	MSP_000590	Holliday junction resolvase RuvX
622357	MSP_000616	GtrA family protein
685001	MSP_000670	helix-turn-helix transcriptional regulator
798190	MSP_000790	ribonuclease D
808000	MSP_000797	tyrosine--tRNA ligase
838049	MSP_000826	N-acetyltransferase
995536	MSP_000969	hypothetical protein
1328572	MSP_001286	hypothetical protein
1404857	MSP_001341	class I SAM-dependent methyltransferase
1425698	MSP_001363	aspartate carbamoyltransferase catalytic subunit
1446788	MSP_001380	molybdopterin adenylyltransferase
1460874	MSP_001396	transglutaminase-like cysteine peptidase
1484526	MSP_001416	radical SAM family heme chaperone HemW
1517521	MSP_001447	excinuclease ABC subunit UvrC
1519688	MSP_001448	CDP-diacylglycerol-glycerol-3-phosphate 3-phosphatidyltransferase
1568328	MSP_001493	Rne/Rng family ribonuclease
1578232	MSP_001502	hypothetical protein
1596437	MSP_001519	hypothetical protein
1621788	MSP_001540	response regulator transcription factor
1645084	MSP_001558	NAD(+) synthase
1646366	MSP_001559	hypothetical protein
1651115	MSP_001563	MarR family winged helix-turn-helix transcriptional regulator
1734895	MSP_001643	secondary thiamine-phosphate synthase enzyme YjbQ
1874613	MSP_001799	hypothetical protein
1944055	MSP_001865	DUF1778 domain-containing protein
1963703	MSP_001883	M48 family metallopeptidase
1977202	MSP_001895	ketoacyl-ACP synthase III
1994069	MSP_001911	cob(I)yrinic acid a,c-diamide adenosyltransferase
2064687	MSP_001987	GNAT family N-acetyltransferase
2081816	MSP_002001	phosphodiester glycosidase family protein
2144514	MSP_002059	uracil-DNA glycosylase
2172507	MSP_002088	FMN-dependent NADH-azoreductase
2275056	MSP_002191	creatininase family protein

2321293	MSP_002232	threonine synthase
2349336	MSP_002261	YqgE/AlgH family protein
2350786	MSP_002263	ribonuclease HI
2404602	MSP_002313	radical SAM protein
2410096	MSP_002322	endonuclease III
2429504	MSP_002341	TonB family protein
2568313	MSP_002454	helicase
2633733	MSP_002501	Glu/Leu/Phe/Val dehydrogenase
2765827	MSP_002634	c-type cytochrome biogenesis protein CcmI
2770190	MSP_002638	DNA cytosine methyltransferase
2876872	MSP_002739	glycoside hydrolase family 25 protein
2930157	MSP_002791	TetR/AcrR family transcriptional regulator
2980352	MSP_002839	MarR family winged helix-turn-helix transcriptional regulator
3087745	MSP_002936	GNAT family N-acetyltransferase
3156139	MSP_003002	CCDC90 family protein
3192383	MSP_003041	chromosome segregation protein SMC
3196736	MSP_003047	type II toxin-antitoxin system RelE/ParE family toxin
3210415	MSP_003057	translation elongation factor 4
3213440	MSP_003059	type II toxin-antitoxin system prevent-host-death family antitoxin
3299590	MSP_003140	nuclear transport factor 2 family protein
3313086	MSP_003154	acetyl-CoA carboxylase, carboxyltransferase subunit beta
3313986	MSP_003155	tryptophan synthase subunit alpha
3340685	MSP_003183	J domain-containing protein
3369421	MSP_003215	hypothetical protein
3447158	MSP_003277	Uma2 family endonuclease
3489026	MSP_003320	class I SAM-dependent methyltransferase
3494370	MSP_003327	3-deoxy-8-phosphooctulonate synthase
3527858	MSP_003356	beta-ketoacyl-ACP synthase I
3536070	MSP_003366	response regulator transcription factor
3537705	MSP_003369	CCDC90 family protein
3548021	MSP_003379	group II truncated hemoglobin
3552301	MSP_003383	hypothetical protein
3586888	MSP_003425	hypothetical protein
3654181	MSP_003504	type II toxin-antitoxin system RelE/ParE family toxin
3717640	MSP_003567	succinyl-diaminopimelate desuccinylase
3762664	MSP_003611	hypothetical protein
3839619	MSP_003684	metalloregulator ArsR/SmtB family transcription factor
3876672	MSP_003718	uracil-DNA glycosylase
3885368	MSP_003728	M48 family metalloprotease
10784	MSP_003975	response regulator transcription factor
253745	MSP_004122	metalloregulator ArsR/SmtB family transcription factor
2515	MSP_004142	hypothetical protein
92349	MSP_004215	putative toxin-antitoxin system toxin component, PIN family
100076	MSP_004221	Fic family protein

Table S7. Expression level of sigma factor-related genes

Locus tag	Annotation	NMS		ANMS	
		RPKM	Percentile	RPKM	Percentile
MSP_002979	RNA polymerase sigma factor RpoH	5173.0	99.3	3773.2	98.8
MSP_003280	RNA polymerase sigma factor RpoD	646.7	94.4	899.7	95.6
MSP_002782	sigma-70 family RNA polymerase sigma factor	328.1	90.1	590.8	93.4
MSP_002788	RNA polymerase factor sigma-54	167.9	84.0	169.8	83.2
MSP_004204	RNA polymerase sigma factor	120.4	80.2	112.7	78.1
MSP_002287	RNA polymerase sigma factor	52.7	69.7	75.3	72.9
MSP_002247	sigma-70 family RNA polymerase sigma factor	20.9	56.5	15.3	47.6
MSP_002707	anti-sigma factor	15.6	51.6	17.7	50.3
MSP_002706	RNA polymerase subunit sigma-24	10.2	44.6	18.5	51.3
MSP_001205	sigma-70 family RNA polymerase sigma factor	5.5	34.9	4.6	29.2
MSP_003214	RNA polymerase sigma factor	5.5	34.8	4.8	29.9
MSP_003982	sigma-70 family RNA polymerase sigma factor	2.8	25.9	3.9	27.2
MSP_001817	sigma-70 family RNA polymerase sigma factor	1.7	20.6	4.0	27.5
MSP_000210	RNA polymerase factor sigma-32	1.3	18.6	1.7	17.8
MSP_003647	RNA polymerase sigma factor	1.0	16.5	3.7	26.5
MSP_001985	RNA polymerase sigma factor	0.3	9.2	0.0	0.0
MSP_004000	RNA polymerase sigma factor	0.0	0.0	0.2	7.0

Table S8. Consensus promoter motifs detected in genes involved in methane metabolism

Gene	TSS position; Locus tag	−35 motif*	*P*-value	−10 motif*	*P*-value	Relevant enzyme or pathway
pmoC2	1226341; MSP_001190	A**TTGACA**AC	1.7E-05	CATGA**TAGGTT**CCGATG	1.3E-02	pMMO
xoxF	3439396; MSP_003271	A**TTGCGC**TT	7.0E-03	TGCGA**CATATT**ATCCCG	1.4E-03	Methanol dehydrogenase
mxaI	2580209; MSP_002464	C**TCGGGA**AA	1.3E-02	-	-	
mxaG	2580830; MSP_002465	G**TCGCGG**CG	1.5E-02	-	-	
mxaJ	2582009; MSP_002466	T**TGGCCG**GG	2.6E-02	-	-	
fae2	3301191; MSP_003143	C**ATGCGA**AA	1.0E-02	-	-	H4MPT pathway
fae3	3427089; MSP_003256	T**TCGCGG**CT	1.2E-02	TCGAC**TCGAAT**TGAAGC	2.4E-02	
fae4	3427859; MSP_003257	C**TTGCCG**AA	3.9E-05	-	-	
mtdB	3300074; MSP_003142	T**TCGCCC**CG	8.7E-03	TGCGG**TCTATG**CGTCTC	1.7E-02	
mch	3430676; MSP_003260	T**TCGCCG**GG	7.6E-03	CTTAG**TAAAGT**CGCGCC	1.2E-02	
fhcB	2551240; MSP_002438	C**TCGACT**TC	1.8E-02	GCTGA**TAGGCA**TAGCGC	3.1E-02	
fdsD	985109; MSP_000960	C**GTGCGC**AC	3.9E-02	TGTGG**CATGAC**CTTGGT	1.1E-02	
fdsA	989005; MSP_000962	G**TCGAGA**GC	1.6E-02	TTCGT**CATTAT**CCAGAA	1.7E-02	
fdsB	989295; MSP_000963	C**TTTCGA**CT	1.3E-02	-	-	
fdsG	991075; MSP_000964	A**TTGATC**CC	1.1E-02	GCGGC**ACTGTT**GGGGCA	4.5E-02	
fhs	2984850; MSP_002844	C**TTGACG**AG	4.9E-05	CTCTC**CATAGC**CACAGG	4.2E-02	H4F pathway
mtkA	2990864; MSP_002849	A**TTGCGG**AG	4.0E-04	ACGTC**TACATC**AATGCA	3.9E-02	Serine cycle
mclA	2995801; MSP_002852	C**TCGCGC**AT	9.1E-03	CTCGC**CACCAT**CAATGA	3.7E-02	
glyA	1752473; MSP_001659	C**CTGCGA**GA	4.2E-02	AGCGT**GATAAG**CGAACC	1.9E-02	
eno	2275944; MSP_002192	T**TCGTCC**CG	3.2E-02	CGGTC**TACAAC**GCGCGC	4.3E-03	
ppc1	972931; MSP_000952	T**TCGCCA**AA	1.5E-03	TCTGC**TATAGC**TGCCCT	7.8E-04	
mdh	1923916; MSP_001848	T**TTTCAG**AC	2.0E-02	CGCGG**TAGCAG**TACATT	2.8E-02	TCA cycle
acnA	2786269; MSP_002654	A**TAGCCG**TA	4.7E-02	TTTCC**TACAAG**ACGCGA	1.2E-02	
icd	926082; MSP_000905	T**TTGCCC**AT	2.8E-03	-	-	
sucD	1849531; MSP_001772	C**TTGATG**TG	9.8E-03	TTCTC**AATAAT**TTGCAT	1.9E-02	
sdhB	1508275; MSP_001436	C**TGGAAC**TC	4.9E-02	GCTGG**AATATG**ACAACC	4.0E-02	
sdhA	1508409; MSP_001437	C**TCGAGA**TG	4.8E-03	-	-	
sdhC	1510922; MSP_001439	A**TTGCGC**AG	1.2E-03	TGCGC**GTTACT**TAAGTC	3.1E-02	
scpA	3777236; MSP_003625	T**TTGCGC**GC	8.3E-03	TTCGC**TAAAAG**CCGGTA	2.6E-03	EMC pathway
mce	203117; MSP_000170	A**TCGAGG**CG	3.5E-03	-	-	
mcd	3154238; MSP_003001	G**TTGCGT**CC	2.2E-02	-	-	
ecm	3216161; MSP_003062	C**TTTCAC**CT	3.3E-02	TGTGT**TATCGA**CCCGGC	3.5E-03	
hbd	2881021; MSP_002744	T**TTGCGC**CG	4.5E-03	-	-	
phaB	1497428; MSP_001426	T**CTGACG**AT	4.4E-02	AGCGC**TAAATA**TATAAA	1.2E-02	
phaA1	1498658; MSP_001427	C**TTGACG**AG	4.9E-05	-	-	
phaA2	3375818; MSP_003218	C**TCGACA**CC	1.6E-03	-	-	
phaC	3768934; MSP_003616	C**GCGACG**CA	3.3E-02	-	-	
phaZ	2852232; MSP_002712	A**TTTCAG**GA	1.6E-02	TTGAC**TATGCT**ATAATT	2.4E-02	

*Sequences located in the −35 and −10 boxes are shown in bold.

Table S9. Stress response related genes highly expressed at 90th percentile RPKM levels

Locus tag	Annotation	Gene	NMS		ANMS	
			RPKM	Percentile	RPKM	Percentile
MSP_000090	molecular chaperone HtpG	*htpG*	353.3	90.4	450.7	91.6
MSP_001854	molecular chaperone DnaK	*dnaK*	8518.0	99.5	11328.9	99.5
MSP_001855	molecular chaperone DnaJ	*dnaJ*	408.8	91.3	454.4	91.7
MSP_003377	chaperonin GroEL	*groL*	3205.8	98.6	4286.2	99.0
MSP_003378	co-chaperone GroES	*groES*	354.2	90.4	450.0	91.6
MSP_001535	nucleotide exchange factor GrpE	*grpE*	677.8	94.7	1073.7	96.2
MSP_002436	J domain-containing protein	-	994.5	96.1	1216.7	96.6
MSP_000205	ATP-dependent zinc metalloprotease FtsH	*ftsH*	2243.3	98.0	2865.5	98.3
MSP_003596	ATP-dependent zinc metalloprotease FtsH	*ftsH*	510.6	92.6	594.3	93.5
MSP_000627	ATP-dependent Clp protease proteolytic subunit	-	1657.3	97.4	1657.4	97.3
MSP_001216	ATP-dependent Clp protease ATP-binding subunit ClpA	*clpA*	1751.7	97.5	1738.6	97.5
MSP_000094	ATP-dependent chaperone ClpB	*clpB*	2188.2	98.0	3058.2	98.5
MSP_001217	ATP-dependent Clp protease adapter ClpS	*clpS*	3367.4	98.7	2878.3	98.4
MSP_003531	ATP-dependent Clp protease ATP-binding subunit ClpX	*clpX*	711.3	94.9	759.9	94.8
MSP_001410	Spy/CpxP family protein refolding chaperone	-	1118.8	96.4	852.7	95.4
MSP_003532	endopeptidase La	*lon*	3279.4	98.7	3854.7	98.8
MSP_003433	heat shock protein HspQ	*hspQ*	2004.3	97.8	1629.9	97.2
MSP_000812	Hsp20 family protein	*hps*	3973.4	99.0	7243.4	99.4
MSP_002601	Hsp20/alpha crystallin family protein	-	3560.0	98.8	5984.3	99.2
MSP_000007	thioredoxin	*trxA*	2809.9	98.4	4608.6	99.1
MSP_000579	superoxide dismutase	*sod*	6479.3	99.4	6221.9	99.3
MSP_003593	glutathione peroxidase	*gpx*	4176.0	99.0	2882.3	98.4
MSP_000565	cytochrome-c peroxidase	*ccp*	378.8	90.8	472.7	92.1
MSP_003247	peroxiredoxin	*prdx*	1403.9	97.0	1769.7	97.5
MSP_000795	peroxiredoxin	*prdx*	566.1	93.5	483.2	92.2
MSP_000899	peptidylprolyl isomerase	-	1268.2	96.8	1490.1	97.1
MSP_000900	peptidylprolyl isomerase	-	1126.8	96.5	1190.3	96.5
MSP_003500	peptidylprolyl isomerase	-	1017.6	96.2	1340.6	96.9

Table S10. Predicted ncRNAs from noncoding TUs using Rfam database

Rfam family	Description	Rfam accession	RNA type	TU ID	E-value
Cobalamin	Cobalamin riboswitch	RF00174	Cis-reg; riboswitch	TU-0580	3.00E-29
Glycine	Glycine riboswitch	RF00504	Cis-reg, riboswitch	TU-1104	1.80E-16
Atu_C6	EcpR1	RF02500	Gene; sRNA	TU-0159	7.30E-17
				TU-0160	7.30E-17
				TU-0161	7.30E-17
				TU-0162	7.30E-17
ar45	Alphaproteobacterial sRNA αr45	RF02347	Gene; sRNA	TU-0903	5.40E-16
				TU-0904	5.40E-16
MIR1858	MIR1858 microRNA precursor family	RF04119	Gene; miRNA	TU-0310	5.80E-09
alpha_tmRNA	Alphaproteobacteria transfer-messenger RNA	RF01849	Gene	TU-0925	1.80E-54

Table S11. List of oligonucleotides used in this study

Oligo name	Sequence (5' - 3')	Note
Universal	AATGATACGGCGACCACCGAGATCTACACTCTTTCCCTACACGACGCTCTTCCGATCT	Forward primer
Index_1	CAAGCAGAAGACGGCATACGAGATCGTGATGTGACTGGAGTTCAGAC	Reverse index primer
Index_2	CAAGCAGAAGACGGCATACGAGATACATCGGTGACTGGAGTTCAGAC	
Index_3	CAAGCAGAAGACGGCATACGAGATGCCTAAGTGACTGGAGTTCAGAC	
Index_4	CAAGCAGAAGACGGCATACGAGATTGGTCAGTGACTGGAGTTCAGAC	
Index_5	CAAGCAGAAGACGGCATACGAGATCACTGTGTGACTGGAGTTCAGAC	
Index_6	CAAGCAGAAGACGGCATACGAGATATTGGCGTGACTGGAGTTCAGAC	
Index_7	CAAGCAGAAGACGGCATACGAGATGATCTGGTGACTGGAGTTCAGAC	
Index_8	CAAGCAGAAGACGGCATACGAGATTCAAGTGTGACTGGAGTTCAGAC	
Index_9	CAAGCAGAAGACGGCATACGAGATCTGATCGTGACTGGAGTTCAGAC	
Index_10	CAAGCAGAAGACGGCATACGAGATAAGCTAGTGACTGGAGTTCAGAC	
Index_11	CAAGCAGAAGACGGCATACGAGATGTAGCCGTGACTGGAGTTCAGAC	
Index_12	CAAGCAGAAGACGGCATACGAGATTACAAGGTGACTGGAGTTCAGAC	
Index_13	CAAGCAGAAGACGGCATACGAGATTGTTGACTGTGACTGGAGTTCAGAC	
Index_14	CAAGCAGAAGACGGCATACGAGATACGGAACTGTGACTGGAGTTCAGAC	
Index_15	CAAGCAGAAGACGGCATACGAGATTCTGACATGTGACTGGAGTTCAGAC	
Index_16	CAAGCAGAAGACGGCATACGAGATCGGGACGGGTGACTGGAGTTCAGAC	
Index_18	CAAGCAGAAGACGGCATACGAGATGTGCGGACGTGACTGGAGTTCAGAC	
Index_19	CAAGCAGAAGACGGCATACGAGATCGTTTCACGTGACTGGAGTTCAGAC	
Index_20	CAAGCAGAAGACGGCATACGAGATAAGGCCACGTGACTGGAGTTCAGAC	
Index_21	CAAGCAGAAGACGGCATACGAGATTCCGAAACGTGACTGGAGTTCAGAC	
Index_22	CAAGCAGAAGACGGCATACGAGATTACGTACGGTGACTGGAGTTCAGAC	
Index_23	CAAGCAGAAGACGGCATACGAGATATCCACTCGTGACTGGAGTTCAGAC	
Index_25	CAAGCAGAAGACGGCATACGAGATATATCAGTGTGACTGGAGTTCAGAC	
Index_27	CAAGCAGAAGACGGCATACGAGATAAAGGAATGTGACTGGAGTTCAGAC	

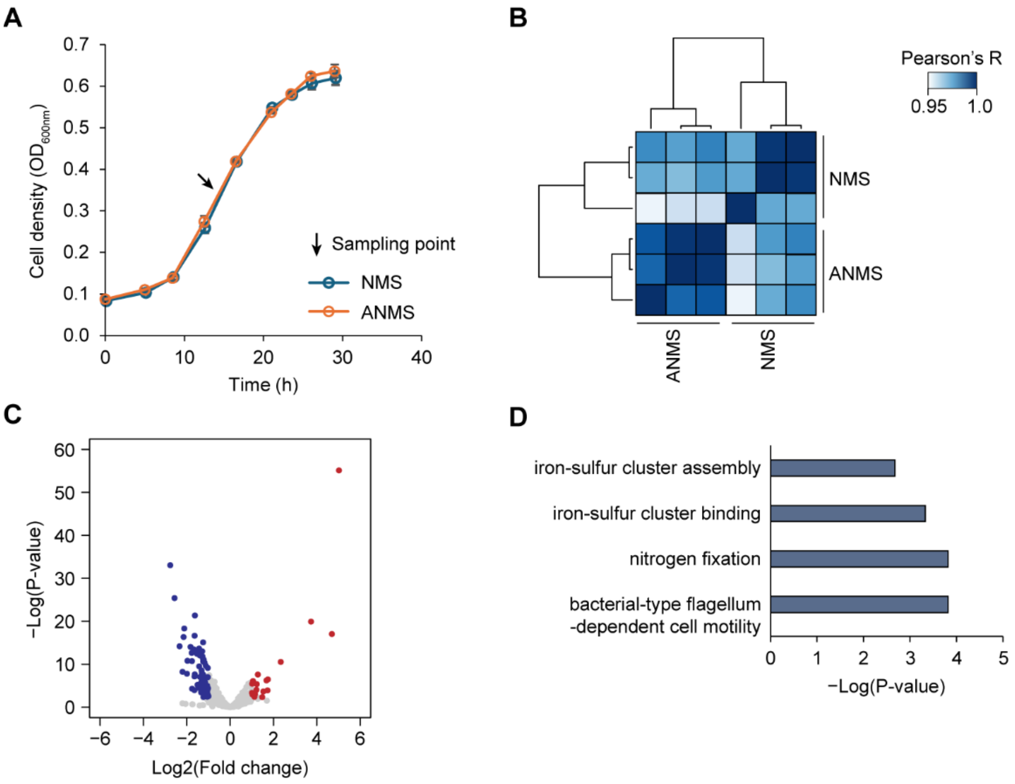

Figure S1. RNA-seq analysis of *M. sporium* 5 grown in ammonium nitrate mineral salts (ANMS) or nitrate mineral salts (NMS) medium with methane. (A) Growth curves of *M. sporium* cultured under methane conditions in ANMS (orange) and NMS (blue) media. The arrow indicates the sampling timepoint for RNA extraction, which corresponds to the mid-exponential growth phase (OD_{600} = 0.35). (B) Pearson's correlation matrix of transcriptome profiles across all condition, shown with hierarchical clustering based on rlog-transformed read counts. (C) Volcano plot of differentially expressed genes between ANMS and NMS conditions. (D) GO term enrichment analysis of 83 downregulated genes under ANMS compared to NMS medium. A Benjamini–Hochberg–corrected $P < 0.05$ was considered statistically significant.

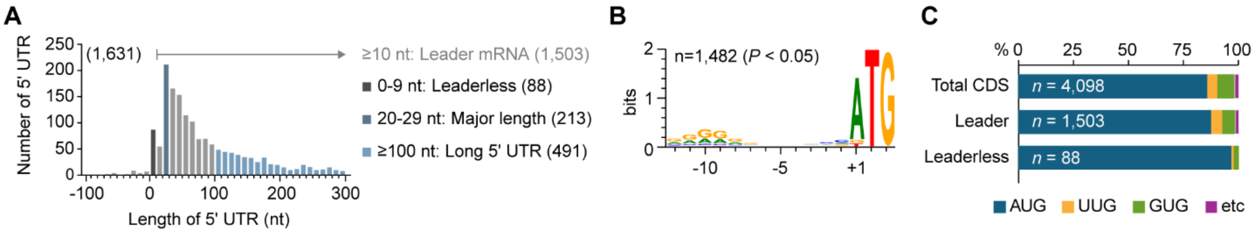

Figure S2. Determination of 5′-UTRs in *M. sporium* 5. (A) Distribution of 5′-UTR lengths associated with primary TSSs. (B) Conserved AG-rich Shine-Dalgarno sequence motif detected in 20-nt sequences upstream of start codons from 1,482 leadered transcripts (5′-UTRs ≥ 10 nt). (C) Start codon usage in total CDSs, leader mRNAs, and leaderless mRNAs.

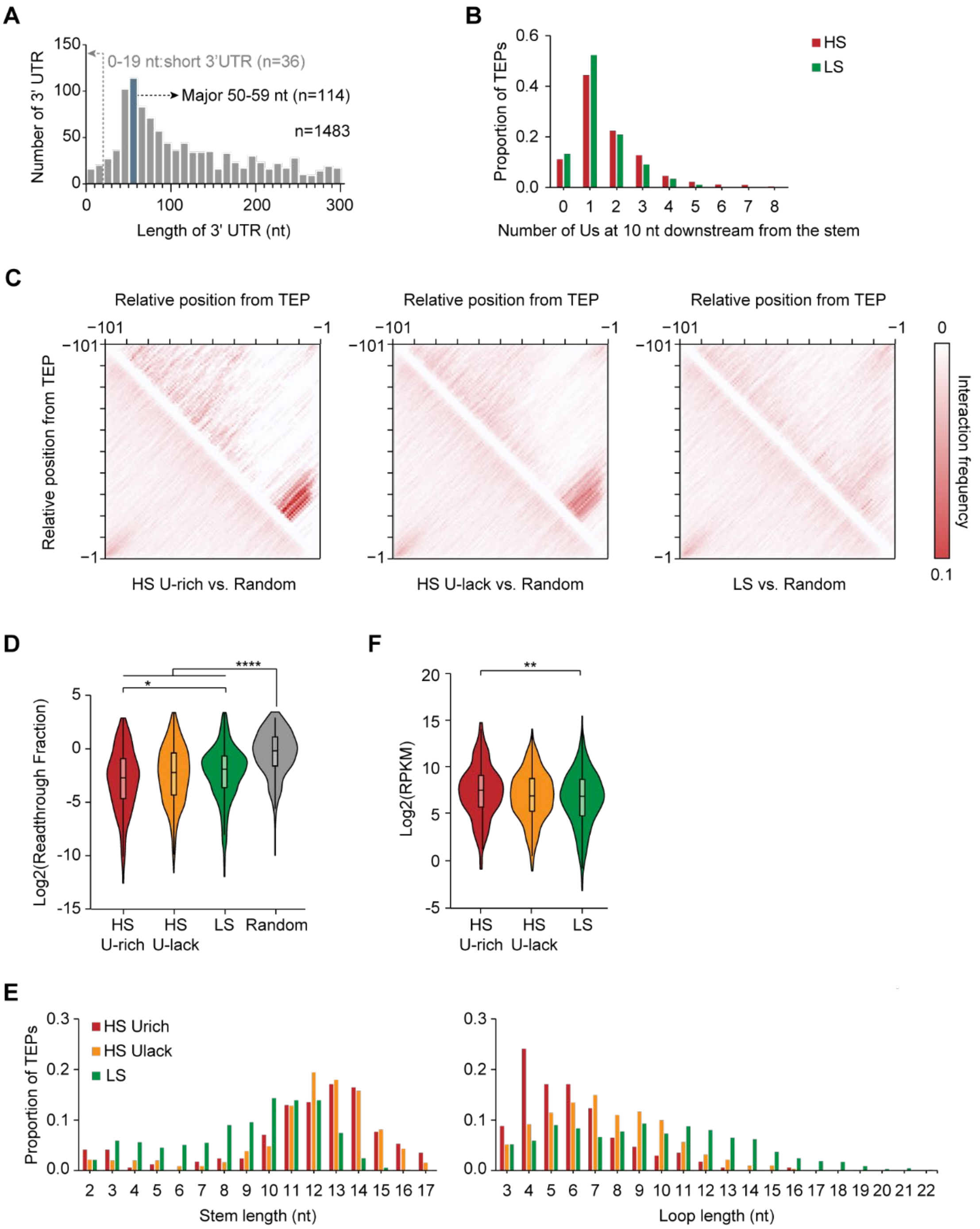

Figure S3. Analysis of three classes of TEPs identified in *M. sporium*. (A) Distribution of 3′-UTR lengths associated with primary TEPs. (B) Distribution of uridine (U) content within the 10-nt downstream of predicted RNA stem-loop structures in highly structured (HS; n = 772) and less structured (LS; n = 711) TEPs. (C) Base-pair interaction frequency within the 100-nt

upstream region of TEPs. HS U-rich (left, upper triangle), HS U-lack (middle, upper triangle), and LS (right, upper triangle) TEP groups were compared with random intergenic positions (lower triangle in all squares). RNA secondary structures were predicted using RNAfold (1) at 30 °C. Interaction frequency was calculated as the ratio of observed base-pair interactions to total possible interactions. Random represents 1,483 randomly selected intergenic positions. (D) Distribution of readthrough fractions, calculated as the average normalized RNA read-count ratio between the upstream (−200 to 0 nt) and downstream (0 to +200 nt) regions of TEPs or random positions. RNA-seq data from NMS conditions were used. Outliers above the 95th percentile were excluded. (E) Distribution of predicted stem and loop lengths for three groups of TEPs. (F) Gene expression levels associated with each TEP group. RPKM values were calculated for each biological replicate under NMS conditions, and the average values were used. *, $P < 0.05$; **, $P < 0.01$; ***, $P < 0.001$; ****, $P < 0.0001$ (Mann-Whitney U test, two-sided).

Supplementary Text

Text S1. Determination of 5′-untranslated regions

The 5′-UTR was defined as the sequence between the primary TSS and start codon of the corresponding gene. This identified 1,503 leader mRNAs with 5′-UTRs ≥ 10-nt, accounting for the majority of transcripts (92%) and 88 leaderless mRNAs (**Fig. S2A**). The median length of the leadered 5′-UTR was 63-nt, with the most frequent length of 20-29 nt. Conserved AG-rich Shine-Dalgarno sequences for ribosome binding were detected upstream of the start codon in 1,482 of leader mRNAs (98.6%, $P < 0.05$), (**Fig. S2B**). Approximately one third of leader mRNAs (n = 491) had long 5′-UTRs (>100 nt), suggesting the potential presence of regulatory RNA structures such as riboswitches that mediate post-transcriptional regulation (2). The abundance of leader mRNAs in *M. sporium* implies that most genes are regulated by regulatory elements in leader sequences that modulate translational efficiency and post transcriptional control (2, 3).

In contrast, leaderless mRNAs initiate translation directly at the start codon without additional translational regulation (4). In *M. sporium*, the identified leaderless transcripts were associated with genes involved in stress response, transcriptional regulators, and toxin-antitoxin systems (**Table S6**), suggesting that leaderless transcription may play a role in rapid adaptation to environmental stress, as reported in other bacteria (5, 6). Notably, ATG was the predominant start codon in leaderless transcripts, which may promote efficient translation initiation in the absence of RBS (**Fig. S2C**). Similarly, ATG was also the most frequently used start codon in leadered transcripts, followed by TTG and GTG as alternative start codons.

Supplementary Methods

Method S1. Sequencing and data analysis

RNA seq library preparation. Ribosomal RNA was depleted using the RiboRid method (7) with custom anti-rRNA oligonucleotide probes targeting *M. sporium* 5 rRNAs. RNA-Seq libraries were prepared from 10–100 ng rRNA-depleted RNA using a TruSeq Stranded mRNA Library Prep Kit (Illumina) and validated with a Qubit 4 fluorometer (Invitrogen) and TapeStation 4150 equipped with a High Sensitivity D1000 Screen Tape (Agilent Technologies).

dRNA-seq library preparation. After treatment of DNase I (NEB) to remove residual genomic DNA in total RNA samples, the RNA samples were divided into TEX-treated (TEX+) and non-treated (TEX−) samples. TEX+ samples were incubated with 1 U of Terminator 5′-Phosphate-Dependent Exonuclease (TEX, Epicentre) in Terminator Reaction Buffer A (Epicentre) with 20 U of SUPERase In RNase Inhibitor (Thermo Scientific) to enrich primary transcripts, while TEX− samples remained untreated in the same reaction mixture. After reaction termination, RNA was purified using RNA Clean & Concentrator-5 Kit (Zymo). To ligate 5′-RNA adaptor, 5′ triphosphate was converted to monophosphate using 20 U of RNA 5′-polyphosphatase (Epicentre) with 20 U of SUPERase In RNase Inhibitor, followed by incubation at 37 °C for 1 h. After RNA purification using RNA Clean & Concentrator-5 Kit, 5 pmol of 5′-RNA adaptor (5′-ACACUCUUUCCCUACACGACGCUCUUCCGAUCU-3′) was ligated using T4 RNA Ligase 1 (NEB) at 23 °C for 2 h 30 min in a reaction mixture containing 2 µL of 10× T4 RNA ligase 1 buffer, 2 µL of 10 mM ATP, 2 µL DMSO, 8 µL of 50% PEG8000, 2 µL T4 RNA Ligase 1, and 1 µL of SUPERase In RNase Inhibitor (20 U/µL). To prevent unintended 3′ end ligation, the

adaptor was dephosphorylated with FastAP Thermosensitive Alkaline Phosphatase (Thermo) to remove 5′ phosphate groups before ligation. Adaptor-ligated RNA was then subjected to rRNA depletion using RiboRid.(7) cDNA was synthesized using random hexamer 3′ overhanging primer (5′-GTGACTGGAGTTCAGACGTGTGCTCTTCCGATCTNNNNNN-3′) and SuperScript III First-Strand Synthesis System (Invitrogen). cDNA libraries were purified using 0.8× volume of Agencourt AMPure XP Beads (Beckman Coulter) and amplified using Phusion High-Fidelity polymerase (Thermo) with indexed primers listed in **Table S11**. Amplification was monitored on the Applied Biosystems^{TM} StepOne^{TM} Real-Time PCR System (Thermo) and stopped before reaching the plateau. The final dRNA-seq libraries were purified with 0.8× AMPure XP beads and validated using Qubit 4 fluorometer and 4150 TapeStation System.

Term-seq library preparation. Term-seq libraries were constructed with modifications to a published protocol.(7, 8) Briefly, 5′-DNA adaptor was ligated to RNA using T4 RNA Ligase 1 (NEB) with 150 pmol amino-blocked 3′-DNA adaptor (5′-p-NNAGATCGGAAGAGCGTCGTGTAGGGAAAGAGTGT-AmMO-3′), followed by RNA purification. The adaptor-ligated RNA was then subjected to rRNA depletion via RiboRid(7) and fragmented at 72 °C for 90 s using RNA Fragmentation Reagent (Ambion), followed by purification with 2.2× AMPure XP Beads. cDNA was synthesized using 10 pmol reverse transcription primer (5′-TCTACACTCTTTCCCTACACG-3′) and SuperScript III First-Strand Synthesis System (Invitrogen). After purification using AMPure XP Beads, 150 pmol cDNA 3′-adaptor (5′-p-NNAGATCGGAAGAGCACACGTCTGAACTCCAGTCAC-AmMO-3′) was ligated using T4 RNA Ligase 1 (NEB) at 23 °C for 8 h, followed by purification using 1.8× AMPure XP Beads. The ligation product was amplified using Phusion High-Fidelity DNA Polymerase (Thermo) with indexed primers (**Table S11**) and stopped before reaching the plateau.

The final library was purified using 0.8× AMPure XP beads and validated using Qubit 4 fluorometer and 4150 TapeStation System.

High-throughput sequencing. RNA-seq libraries were sequenced on an Illumina MiSeq platform (2 × 75 bp). dRNA-seq and Term-seq libraries were sequenced on an Illumina NextSeq 1000 platform (1 × 100 bp). Each library generated 3.8–9.1 million reads. Sequencing data were processed and mapped to the genome of *M. sporium* 5 using CLC Genomics Workbench 6.5.1 (Qiagen), yielding 55.1–95.9% uniquely mapped reads with 46.8–143.9-fold genomic coverage (**Table S2**).

Sequencing data processing. First, low-quality reads and adaptor sequences were trimmed from raw sequencing data (quality limit, 0.05; maximum ambiguous nucleotides, 2). For dRNA-seq, reads < 25 nt after trimming were discarded. For Term-seq, two random nucleotides added during adaptor ligation were removed from both ends and reads < 15 nt were discarded. Term-seq reads were then inverted, as the sequencing was generated in the reverse direction. Finally, resulting trimmed reads were mapped to the reference genome (mismatch cost, 2; insertion cost, 3; deletion cost, 3; length fraction, 0.9; similarity cost, 0.9; and ignore nonspecific match), and only uniquely mapped reads were retained.

Identification of transcription start sites. TSSs were determined from TEX+ reads and curated against TEX− and RNA-seq data, as described previously with slight modifications.(9) Briefly, initial TSS peaks within 100 nt were clustered, and adjacent peaks in each cluster were sub-clustered based on a standard deviation (< 10) to select a local maximum peak as the TSS in each sub-cluster. Clusters with ≥3 read counts were considered, and the TSS peaks with maximum read counts were selected as potential TSSs. To refine TSS selection, assigned TSSs were

compared with TEX− data, retaining only those present within ±5 nt. Final TSSs were manually inspected against RNA-seq profiles. TSSs were categorized based on their genomic positions relative to annotated genes. Among the TSSs located from 300 nt upstream to 100 nt downstream of the 5′-end of respective gene, those with the highest peak intensities (read counts) were classified as primary (P), while the others as secondary (S) TSSs. TSSs located within annotated genes or on the opposite strand were classified as internal (I) or antisense (A), respectively. TSSs that did not fall into either of these categories were classified as intergenic (N). The TSS results obtained from two conditional libraries were merged within ±5 nt and designated as total TSSs, which are listed in **Data S2**.

Identification of transcript 3′-end positions. TEPs were identified from Term-seq reads using a similar clustering approach (10, 11) Initial peaks were clustered as described for TSS peak clustering. Low-intensity peaks <10 read counts (90th percentile) and peaks absent in all three biological replicates were discarded. Further filtering was applied based on peak enrichment, selecting peaks with a z-score above 4. Only reproducible peaks with the highest read count across replicates were retained as TEPs, followed by manually inspected against RNA-seq profiles. TEPs were classified based on genomic positions relative to annotated genes. Among the TEPs located less than 300 nt downstream of the respective gene, TEPs with the highest read counts were classified as primary (P), while the others as secondary (S) TEPs. TEPs located on the opposite strand were classified as antisense (A). TEPs that did not fall into either of these categories were classified as intergenic (N). Internal TEPs within coding regions were excluded. For TEPs located between 100 nt downstream of the primary TSS and the 5′-end of the associated gene, they were classified as *cis*-regulatory (C) TEPs. The minimum distance was set to 100-nt to ensure proper formation of terminator structure. The TEP results obtained from two

conditional libraries were merged within ±5 nt and designated as total TEPs, which are listed in **Data S2**.

References

1. Lorenz R, Bernhart SH, Höner zu Siederdissen C, Tafer H, Flamm C, Stadler PF, Hofacker IL. 2011. ViennaRNA Package 2.0. Algorithms for molecular biology 6:1-14.
2. Breaker RR. 2011. Prospects for riboswitch discovery and analysis. Mol Cell 43:867-79.
3. Shine J, Dalgarno L. 1974. The 3´-terminal sequence of Escherichia coli 16S ribosomal RNA: complementarity to nonsense triplets and ribosome binding sites. Proceedings of the National Academy of Sciences 71:1342-1346.
4. Udagawa T, Shimizu Y, Ueda T. 2004. Evidence for the translation initiation of leaderless mRNAs by the intact 70 S ribosome without its dissociation into subunits in eubacteria. J Biol Chem 279:8539-46.
5. Vesper O, Amitai S, Belitsky M, Byrgazov K, Kaberdina AC, Engelberg-Kulka H, Moll I. 2011. Selective translation of leaderless mRNAs by specialized ribosomes generated by MazF in Escherichia coli. Cell 147:147-57.
6. Leiva LE, Katz A. 2022. Regulation of Leaderless mRNA Translation in Bacteria. Microorganisms 10.
7. Choe D, Szubin R, Poudel S, Sastry A, Song Y, Lee Y, Cho S, Palsson B, Cho BK. 2021. RiboRid: A low cost, advanced, and ultra-efficient method to remove ribosomal RNA for bacterial transcriptomics. PLoS Genet 17:e1009821.
8. Dar D, Shamir M, Mellin JR, Koutero M, Stern-Ginossar N, Cossart P, Sorek R. 2016. Term-seq reveals abundant ribo-regulation of antibiotics resistance in bacteria. Science 352:aad9822.
9. Jeong Y, Kim JN, Kim MW, Bucca G, Cho S, Yoon YJ, Kim BG, Roe JH, Kim SC, Smith CP, Cho BK. 2016. The dynamic transcriptional and translational landscape of the model antibiotic producer Streptomyces coelicolor A3(2). Nat Commun 7:11605.
10. Lee Y, Lee N, Jeong Y, Hwang S, Kim W, Cho S, Palsson BO, Cho B-K. 2019. The transcription unit architecture of Streptomyces lividans TK24. Frontiers in Microbiology 10:2074.
11. Hwang S, Lee N, Choe D, Lee Y, Kim W, Jeong Y, Cho S, Palsson BO, Cho B-K. 2021. Elucidating the regulatory elements for transcription termination and posttranscriptional processing in the Streptomyces clavuligerus genome. Msystems 6:10.1128/msystems. 01013-20.
